# Sixty Degrees of Solutions: Field Techniques for Human–Jaguar Coexistence

**DOI:** 10.3390/ani15091247

**Published:** 2025-04-28

**Authors:** John Polisar, Rafael Hoogesteijn, Almira Hoogesteijn, Diego Francis Passos Viana, Skarleth Johana Chinchilla Valdiviezo, Carlos Valderrama Vásquez, Allison Loretta Devlin, Ranni José Arias Herrera, Margaux Babola, Frederick Bauer, Ivonne Cassaigne Guasco, Chia Yu Chang, Daniel Corrales Gutiérrez, J. Antonio De la Torre, Wezddy Del Toro-Orozco, Aline Kotz, Duston Larsen, Nicolás Lodeiro Ocampo, Daniel Monzón, Carmen Angélica Morante Ascanio, Ricardo Daniel Ortiz-Hoyos, Pablo Gastón Perovic, Grasiela Edith de Oliviero Porfirio, María Fernanda Puerto Carrillo, Paul Raad, Thiago Reginato, Yina Paola Serna, Claudio Sillero-Zubiri, Laura Villalba, Armand Ziller

**Affiliations:** 1 Center for Biodiversity, Department of Environment and Development, Zamorano University Pan-American Agricultural School, Tegucigalpa P.O. Box 93, Honduras; 2 Panthera, New York, NY 10018, USA; rafhoogesteijn@gmail.com (R.H.); adevlin@panthera.org (A.L.D.); cchang@wcs.org (C.Y.C.); dcorrales@panthera.org (D.C.G.); rortiz@panthera.org (R.D.O.-H.); yserna@panthera.org (Y.P.S.); 3 Sierra National Forest, Clovis, CA 93611, USA; 4 Wildlife Conservation Society, Bronx, NY 10460, USA; 5 Independent Veterinary Doctor (DVM) & Jaguar/Livestock Conflict Resolution Consultant, Campo Grande 79037-072, MS, Brazil; 6 Human Ecology Department, Merida Unit, Cinvestav, Ciudad de México 97310, Mexico; almirahoo@cinvestav.mx; 7 Instituto Homem Pantaneiro—IHP, Gestor do Programa Felinos Pantaneiros, Corumbá 79300-010, MS, Brazil; vianadiego26@gmail.com (D.F.P.V.); grasi_porfirio@hotmail.com (G.E.d.O.P.); 8 Center for Biodiversity, Zamorano University, Tegucigalpa P.O. Box 93, Honduras; chinchilla.skarleth@gmail.com; 9 WebConserva, Bogotá 111211, Colombia; carlos.valderrama@webconserva.org; 10 Desarrollo Forestal Sustentable (DEFORSA), San Carlos 2201, Cojedes, Venezuela or ariasranni@gmail.com (R.J.A.H.); cmorante50@gmail.com (C.A.M.A.); 11 Human Initiatives for Animals (HISA), French Office for Biodiversity, Cayenne 97300, French Guiana, France; margaux.babola@hisaproject.org (M.B.); armand.ziller@hisaproject.org (A.Z.); 12 Wildlife Conservation Society—Paraguay, Asunción 1208, Paraguay; fbauer@wcs.org (F.B.); lvillalba@wcs.org (L.V.); 13 Primero Conservation, Rancho la Herradura, México City 04930, Mexico; icassaigne@yahoo.com; 14 Wildlife Conservation Society, Belize City, Belize; 15 Programa Jaguares de la Selva Maya—Bioconciencia A.C., México City 10400, Mexico; adelatorre.jsm@gmail.com (J.A.D.l.T.); monzondaniel1996@gmail.com (D.M.); 16 Ecology and Conservation of Amazon Felids Research Group, Instituto de Desenvolvimento Sustentável Mamirauá, Tefé 69552-225, AM, Brazil; biowezddy@gmail.com; 17 Center for Integrative Conservation Research & Warnell School of Forestry and Natural Resources, University of Georgia, Athens, GA 30602, USA; 18 Projeto Onças do Iguaçu-Instituto Pró Carnívoros, Parque Nacional do Iguaçu, Foz do Iguaçu 85859-899, PR, Brazil; aline_kotz@hotmail.com (A.K.); thiago_fln@yahoo.com.br (T.R.); 19 Integrated Ranch, Tourism, and Wildlife Refuge Operation—San Miguelito, Bolivian Chaco/Chiquitania, Santa Cruz de la Sierra, Bolivia; duston1@gmail.com; 20 Fundación Red Yaguareté, Cuenca, 1539 PB4, Buenos Aires CP 1406, Argentina; nicolas@redyaguarete.org.ar; 21 Vicerectadora de Infrastructures y Processos Industriales, Universidad Nacional Experimental de los Llanos Occidentales “Ezequiel Zamora”—UNELLEZ, San Carlos 2201, Cojedes, Venezuela; 22 Administración de Parques Nacionales—Dirección Regional Noroeste, Salta 4400, Argentina; pgperovic@gmail.com; 23 Jaguars in the Fringe, Vaqueros, Salta 4401, Argentina; 24 Instituto Venezolano de Investigaciones Científicas (IVIC) y Proyecto Sebraba, Altos de Pipe, San Antonio de los Altos 1204, Estado Miranda, Venezuela; maripuerto@gmail.com; 25 Fazenda Ipiranga, Estr. Transpantaneira, KM-10-Zona Rural, Poconé 78175-006, MT, Brazil; paulraadc@gmail.com; 26 Wildlife Conservation Research Unit, Department of Biology, University of Oxford, Oxford OX1 2JD, UK; claudio.sillero@biology.ox.ac.uk

**Keywords:** jaguars, depredation, human–jaguar coexistence, anti-depredation strategies, carnivore conservation, carnivores, livestock

## Abstract

The current range of the jaguar (*Panthera onca*) spans sixty degrees of latitude across eighteen countries in the Western Hemisphere and covers approximately 7,000,000 km^2^. Throughout this geographical breadth, jaguars represent an essential component of native biological diversity, but conflict revolving around real and perceived jaguar depredation on livestock is a factor in jaguar mortality. We developed a structured questionnaire to evaluate the effectiveness of anti-depredation strategies from northern Mexico to Argentina, collecting data from 11 countries and 248 livestock operations, 194 with efficacy metrics, and 24 with benefit–cost ratios (value of the livestock losses averted/cost of the intervention). Using coarse categories, 11 intervention types were tested. Techniques effectively reducing livestock losses were documented across the entire livestock operation size (2–130,000 ha, 5–30,000 head) and biome spectrum. While the techniques varied in complexity and required levels of investment, successful reductions in depredation were achieved at all levels. We conclude that anti-depredation strategies are highly effective, and when benefits are evaluated, they surpass costs, sometimes substantially. Given the proven efficacy and cost-effectiveness of the techniques described in this paper, we advocate for broader application across the species range to increase tolerance towards jaguars and a more effective human–jaguar coexistence.

## Simple Summary

The jaguar spans about 7,000,000 km^2^ across the Americas. Livestock predation by jaguars often leads to retaliatory killings, but methods to reduce the frequency of these events have been developed and tested. We surveyed 248 livestock operations from northern Mexico to Argentina and evaluated the success of anti-depredation strategies implemented across 194 of those operations. These strategies necessitated varying levels of investment, but all achieved a notable reduction in depredation and, in most cases, were cost-effective. Anti-depredation strategies are effective, can be adapted to local needs, and merit wider application.

## 1. Introduction

Large carnivores have impressed and intimidated human societies for thousands of years, but their survival is now one of our most pressing conservation challenges [1,2]. Human expansion has led to a decline in their populations worldwide due to habitat loss, fragmentation, degradation, and direct killing [3,4,5,6]. Carnivores play a crucial role in ecological processes and ecosystem structure and function by influencing the density and dynamics of prey species, which are typically primary consumer herbivores [2,3,4,5,6,7]. Large felids can play a role in vertebrate and invertebrate diversity and ecosystem services [8,9,10]. Their position at the top of the food chain makes them an important indicator of the status of biodiversity conservation [11]. Anthropogenic habitat fragmentation disrupts the behavior of carnivores with large home ranges [12,13] and can lead to increased conflicts with humans [14]. On a worldwide scale, inadequate livestock management can contribute to such conflicts and thus pose a significant threat to the survival of large felids and an issue to address [2,15].

The jaguar (*Panthera onca*) is the largest cat in the Americas and fits all the above-described global trends. Despite occupying around 50% of its original range, with sub-populations occurring from the northern Mexico–United States border area to the north of Argentina, the species is overall in decline. Jaguar habitats encompass multiple biomes, ranging from semi-xeric cactus-rich scrub forests to the flooded forests of the Amazon, spanning approximately 60° of latitude [16,17,18]. In 2020, the Jaguar 2030 Roadmap [19] estimated the remaining jaguar habitat at 7 million square kilometers. A team of experts estimated that, in 2023, there was 14% less jaguar habitat in South America than in 2015 and 25% less than in 2000 [20]. These decreases in habitat due to the expansion of human dwellings and agriculture emphasize the need for effective tools for coexistence.

Habitat connectivity for jaguars is still relatively intact in South America’s central Amazonian and Guiana Shield areas. While connectivity is still favorable in those and several other significant areas within the jaguar’s range, even in the most extensive extant blocks of jaguar habitat, fragmentation is increasing rapidly. Outside of the few truly vast areas of wild habitat, most sub-populations are endangered [21], and overall, the range-wide connectivity between fragmented jaguar populations is extremely tenuous and at risk [20]. Despite isolated success stories, the range reduction has been severe in many areas, and the trends show little sign of reversal. The jaguar has been eliminated from 77% of its historical range (or more) in Mesoamerica [22,23,24]. The jaguar is classified as “Near Threatened” by the IUCN Red List [25] and listed on Appendix I of CITES (www.cites.org). This status does not accurately reflect its imperiled status in most range states. On national levels, the jaguar is listed as “Extinct in the Wild” in two countries, “Critically Endangered” in four countries, “Endangered” in seven countries, “Vulnerable” in four countries, and “Near Threatened” in two countries [26,27].

The main threats to jaguar populations are habitat loss [28,29,30], reduced availability of natural prey [30,31,32], and direct killing, often due to conflicts with livestock [4,33,34,35,36]. Another factor contributing to direct fatalities is the trade of body parts, which varies in scale and characteristics across the species’ range [25,37,38]. The interactions of habitat loss, reduced natural prey, and direct killing often combine synergistically to drive local extinctions. For example, 85% of the jaguar habitat in the Atlantic Forest biome has been lost, with only 7% remaining in good condition. Within that, jaguars now only occupy 2.8% of their previous range. The primary causes of jaguar declines in that area were habitat loss and fragmentation, which drove increased proximity of humans, livestock, and jaguars. Direct killing became a critical threat in the remaining habitat [39].

Although habitat loss exerts a prominent role in the decline of the jaguar population, human–jaguar conflicts caused by livestock depredations significantly contributed to the contraction of the historical range of distribution of the species from Mexico to Argentina [40,41]. Human–jaguar conflicts focused on livestock were the primary factor in eradicating jaguars from the northernmost portion of their range in the United States [42], with similar dynamics driving range reduction on the southern edge of their distribution in Argentina [43]. The need for tools for successful coexistence has been urgent for decades.

Scientific observations on the jaguar depredation of livestock started in the 1980s [41,44]. During the first scientific study focused on jaguars and their prey [45,46], which took place on a ranch, the authors were forced to change the study area because ranch workers were killing the study animals. Strategies to reduce these conflicts were introduced in the early 1990s [47]. By the mid-1990s and early 2000s, knowledge and recommendations for lowering human–jaguar conflicts and improving coexistence increased [34,48,49].

Large-scale analyses of the status of jaguars started in 1999 [22]. These were accompanied by wide-ranging information sharing [18,50,51], which encouraged and facilitated discussions about the causes and solutions for human–jaguar conflicts [4,33]. These also include manuals that provide tools to reduce jaguar attacks on livestock [52,53].

Formal efforts to create comprehensive jaguar conservation strategies started in March 1999 when 29 jaguar experts from ten countries joined to evaluate the status of the jaguar and develop a detailed database describing the geographic distribution of jaguars and knowledge about them at the end of the twentieth century [51]. In 2006–2007, the Mesoamerican Jaguar Corridor concept was introduced [54,55]. Another significant milestone in the conservation of jaguars was the inaugural range-wide meeting on jaguar conservation at the United Nations in March 2018 [19]. Delegates from 14 jaguar range states joined the meeting, which marked the beginning of the multi-institutional and multi-governmental Jaguar 2030 conservation initiative, which includes the Jaguar 2030 Roadmap for the Americas [19]. The same momentum facilitated the implementation of the World Wildlife Fund’s Jaguar Strategy 2020–2030 [56]. The Jaguar 2030 initiative currently involves most jaguar range states, various United Nations agencies, several international conventions, numerous NGOs, and local communities. This high-level initiative aims to increase attention on jaguars to create tangible conservation impacts across the jaguar range, including where humans and jaguars coexist.

At the local level, there often needs to be more clarity between how farmers and ranchers perceive the impact of jaguar depredation on livestock and the reality of those impacts [33]. However, actual losses can be severe, especially for small-scale operators. There is no doubt that, historically, direct killing associated with actual or perceived conflict contributed to the decline of the jaguar range and that it continues to do so today. Killing jaguars may be illegal in almost every country in the jaguar’s range [27], but it is happening somewhere, to this very day, and is usually associated with livestock. While we recognize that habitat loss is the most significant threat across the jaguar range, we are also aware that (i) habitat loss generates a synergy that exacerbates conflicts with livestock, (ii) the direct killing of jaguars associated with livestock remains an enormous threat, and (iii) tools to reduce jaguar attacks on livestock have been tried and tested in select locations across much of jaguar range, and they now need to be upscaled.

Tools to reduce jaguar attacks on livestock have been tested in multiple countries, cultures, biomes, and contexts. Those tools may also reduce puma (*Puma concolor*) attacks on livestock. Building on the opportunities created by large-scale jaguar conservation efforts, we conducted a comprehensive study to assess the effectiveness of anti-depredation strategies (ADS). Our primary focus is jaguar conservation, but the findings also relate to puma. Our study aimed to determine which ADS have been tested, where they have been tested, the specific contexts in which they were tested, and their effectiveness in reducing livestock and financial losses. We also sought to identify the most effective techniques for different contexts. Whereas Khorozyan and Waltert (2019) [57] conducted a global review of the efficacy of interventions used to protect livestock from wild cats, including jaguars, in this study, we examine the tools used across the jaguar range in greater detail and across a broader spectrum of livestock operations. Our study compiles data from practitioners across eleven countries, encompassing various biomes and livestock operations, from small to large. It incorporates information from private, community, and indigenous operations, extending from northern Mexico to Argentina. This marks the first comprehensive evaluation of the effectiveness of jaguar anti-depredation tools at this scale and depth.

## 2. Materials and Methods

A core team developed a detailed questionnaire to distribute among a large group of jaguar biologists and livestock operators from northern Mexico to northern Argentina who possess field experience in techniques designed to reduce jaguar attacks on livestock. The questionnaire was developed iteratively by the following experts: John Polisar (experience in this subject from 1996 to 2020 across Central America and parts of South America); Rafael Hoogesteijn (experience from 1987 to 2025 in South America and areas of Central America); and Almira Hoogesteijn (1991–2025) with experience in parts of South America and Mexico. Over 90 years of cumulative experience working in and with non-governmental organizations, universities, and particularly within ranches and farms contributed to the original design of the questionnaires, which were then shared with the broader circle of participants. The questionnaire covered 12 pages and included significant details in every subsection. This questionnaire was divided into several sections [(Supplementary Materials S1): Study Participants and Study Area; Site Characteristics; Livestock Characteristics; Pasture Characteristics; Livestock Operation Management; Purpose of Livestock; Characteristics of Attacks; Methods for Deploying Attack-Reducing Strategies; Employed Methods and Their Features; Results (Effectiveness of the Implemented Methods); Discussion and Conclusions Drawn from Participants’ Experiences]. Summaries of site-specific responses are available in the Supplementary Materials S2.

All participants represented either an individual site or a cluster of sites (some represented many) with relevant information on the study topic. The questionnaire was shared with these experienced experts but did not constitute a classical “expert elicitation” involving opinions, projections, or conjectures. Instead, it served as a tool among experienced experts to gather and organize available information to standardize results. Participation signified consent, which was solicited and granted by all authors. With each 17-site cluster team listed as co-authors, anonymity was not required. An additional four known sites/site clusters were invited but did not participate.

The livestock types and classes that we included in our analyses were bovids (cattle and Asiatic water buffalo (*Bubalus bubalis*), hereafter buffalo), equids (horses, mules, donkeys), ovids, caprids, and suids (sheep, goats, and pigs, respectively). We did not include domestic dogs or birds. While many domestic dogs are lost to jaguars and some domestic birds, the management techniques to reduce those attacks and losses differ from those used for large grazing and browsing domestic herbivores. In contrast, there is cohesion among the practices deployed for the domestic animals that we focused on.

We extracted methods featuring clear efficacy metrics from the completed questionnaires to reduce encounters with jaguars and pumas from 194 livestock operations, representing 77% of the 248 operations analyzed. The remaining 23% (57) reported on feline attacks and their characteristics but did not provide well-defined metrics to evaluate the success of the implemented strategies. In the 194 operations that detailed the impacts of the interventions applied in the field, we investigated the specific aspects of decreased livestock losses resulting from these methods.

Only 24 livestock operations had sufficiently detailed information to allow for an examination of benefit/cost ratios as a cost-effectiveness test. We used data from six ranches in the Paraguayan Chaco [58], 16 in the Colombian Llanos [59], and two ranches in the Brazilian Pantanal to examine the ratio between the investments that ADS required and the benefits in terms of livestock losses averted. The ratio was obtained by dividing the economic benefit (the value of the livestock not preyed upon) by the intervention cost. In all these cases, the losses averted figures were based upon either before–after data (n = six Paraguayan ranches) or strict controls (n = sixteen ranches in Colombia and two in Brazil). A ratio greater than 1.0 indicated that the intervention was financially advantageous, meaning that the strategy generated an economic benefit that exceeded its cost. The total cost of implementing electric fences and night enclosures (and/or other techniques) was calculated individually for each property. The expenses included materials and labor for installation and maintenance. The economic benefit was assessed based on the value of the livestock that were no longer preyed upon after implementing the strategies. This calculation was made by considering the historical predation rates for those specific areas of each ranch, or control areas that experienced losses, and the market value of the livestock that were protected (not lost to depredation). More generalized evaluations of benefits yielded by interventions were available for the study area in Mexico’s Selva Lacandona [21], Nicaragua’s Mosquitia [60], and Casanare Llanos [59]. This study concentrated strictly on investments made in ADS and the returns yielded in livestock losses averted.

## 3. Results

**Figure 1:**
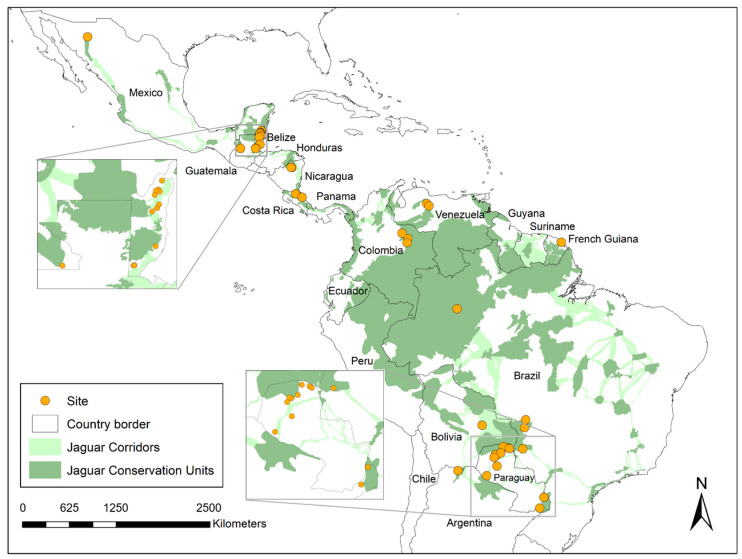
Map of the study area, including the Jaguar Corridor (Jaguar Conservation Units, dark green; Jaguar Corridors, light green) with points indicating the general location of participating livestock operations (n = 248 total). The following eleven countries were represented within the dataset (listed alphabetically): Argentina (n = 26); Belize (n = 12); Bolivia (n = 1); Brazil (n = 86); Colombia (n = 22); Costa Rica (n = 13); French Guiana (n = 4); Mexico (n = 31); Nicaragua (n = 43); Paraguay (n = 9); and Venezuela (n = 1).

**Figure 2:**
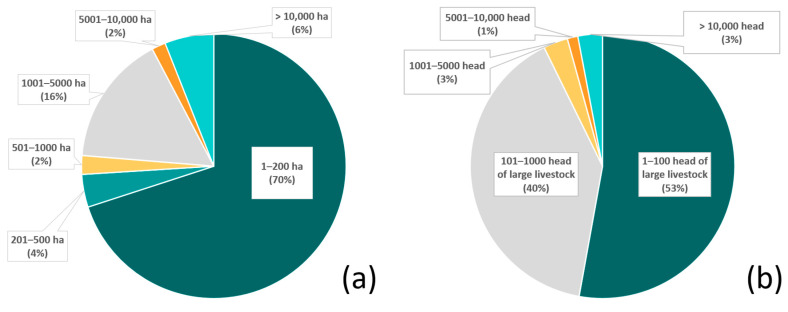
The percentage of livestock operations included in the study classified by (**a**) property size (hectares, ha; data available for n = 176 total properties) and (**b**) herd size, estimated by number of head of large livestock (data available for n = 229 properties).

**Figure 3:**
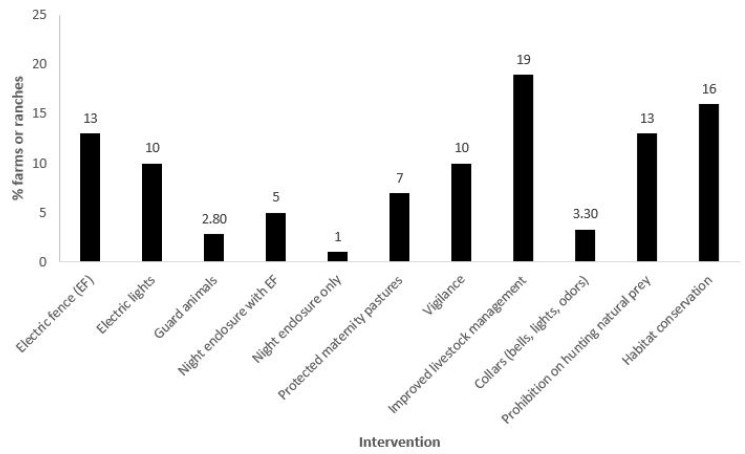
The percentage of livestock operations (n = 248 total, independent of size and number of animals) included in the study that used at least one of the following 11 intervention types to mitigate jaguar/livestock conflict: electric fencing (EF); electric lights; guard animals (e.g., buffalo, creole cattle, donkeys, dogs); night enclosure with EF; night enclosure only; protected maternity pastures (or pasture for young livestock, near human dwellings); vigilance (day or night); improved livestock management (e.g., improved nutrition or herd health practices, excluding livestock from forested areas); equipping livestock with deterrence collars (e.g., bells, bells and lights, odors); prohibition on hunting of natural prey species; and habitat conservation.

The questionnaires were completed in 11 countries (Figure 1: Mexico, Belize, Nicaragua, Costa Rica, Colombia, Venezuela, French Guiana, Bolivia, Brazil, Paraguay, and Argentina). A total of 45 individuals (including members from 17 site/site cluster teams) completed the questionnaire. Due to space constraints, only 30 individuals are listed as authors, while the remainder are acknowledged separately. Four more sites and site clusters were invited but did not fully engage. This included representatives from non-governmental organizations in Guatemala and Panama and two more ranches in Bolivia. No reasons were provided in those cases, but time constraints and/or data characteristics are inferred as likely reasons for not fully engaging. The livestock operations ranged from 2 hectares (ha) to 130,000 ha and five to 30,000 head of livestock (Figure 2a,b). This included private, Indigenous, and community livestock operations. We sampled several biomes, including the following: semi-arid Sonoran desert in northern Mexico; lowland and montane humid forests in southern Mexico, Belize, Nicaragua, and Costa Rica; Los Llanos (seasonally flooded lowland savanna–forest mosaics) in Colombia and Venezuela; Amazon and Guianan Shield forests (Brazil and French Guiana); Chaco (low stature tropical dry forest) in Paraguay; a Chaco-Chiquitania (higher stature semi-deciduous forest) transition zone in Bolivia; Yungas (montane forests) in Argentina; Atlantic Forest in Argentina; and the Pantanal (a seasonally flooded savanna–forest mosaic) in Brazil. A summary of reports is provided in Supplementary Materials S2. Using coarse categories, 11 intervention types were tested (Figure 3).

Approximately 92% of the livestock operations were between 2 and 5000 ha in size; within those, 70% were between 2 and 200 ha (Figure 2a). Approximately 93% of the operations had between 5 and 1000 head of livestock, with 53% less than 100 head (Figure 2b). Although some operations utilized a single ADS, it was typical for multiple ADS to be deployed. Methods included tighter livestock management (19% of the livestock operations), bans on hunting of natural prey or restoration of natural prey (13%), electric fences (13%), electric lights (10%), increased vigilance (10%), a variety of special protections for young animals (7%), and night enclosures (6%), all of which totaled 78% of the methods deployed. The protection of natural habitats (which also preserve natural prey bases) was practiced by 16% of the operations. Approximately 6% had experimented with collars (with bells and or lights or odors) and guard animals (Figure 3).

Among the 194 livestock operations that measured the efficacy of the methods deployed, there were examples of successes from both the largest and smallest operations (Table 1). The largest operation in the study (30,000 head of cattle in 130,000 ha in the Brazilian Pantanal) deployed electrified night enclosures for mother cows and calves, electric lights in pastures (mothers and calves), bells on cows, fences that excluded livestock from the forest, a complete ban on hunting, and forested corridors for wildlife. On that ranch, depredation losses in areas without electric fences were 5.92 to 22.8 times greater than those where ADS were deployed. The smallest operations in the study (80% of herds under 25 head) were in remote Indigenous territories in the humid forests of the Nicaraguan Mosquitia. A combination of improved husbandry methods in these operations included improved nutrition/pastures, exclusion from the forest, enhanced vigilance, and a ban on hunting of key prey species; these interventions resulted in a 100% reduction in losses to depredation. In another remote setting with small Indigenous and community operations in Mexico’s Selva Lacandona (averaging 90 cattle and 50 sheep), night enclosures achieved a 97% reduction in losses of sheep, and electric-fenced maternity pastures achieved an 86% reduction in losses of vulnerable cattle classes.

Techniques effectively reducing losses were documented across the entire operation size and biome spectrum (Table 1). We illustrate this with a few select examples. In the Chaco of Paraguay, combinations of methods (electric lights, electric fence, improved husbandry, exclusion from forests, cleaner pastures, controlled birthing seasons, secure maternity pastures) deployed among ranches ranging from 1000 to 38,627 ha and up to 17,500 cattle resulted in a 100% reduction in losses. In operations ranging from 101 to 500 cattle in the Colombian Llanos, combinations that included maternity paddocks (electric-fenced pastures for mother cows and calves), electric-fenced night enclosures for vulnerable young classes, exclusion from the forest, increased vigilance, strategic placement of pastures and water sources, a partial ban on hunting and deforestation, and creole Sanmartinero bulls introduced to Cebu herds resulted in a 100% reduction of losses in 14 ranches and, in six ranches, a reduction of losses less than 100% but ≥75%. In a large community herd of 1300 cattle across 80,000 ha in Argentina’s Yungas (montane forests), electric fences controlled depredation with 100% effectiveness, as did pastures with electric lights and near houses. In medium-sized operations in the lowland humid forests of Costa Rica (average 520 cattle), electric fences controlled (reduced by 100%) depredation on 12 ranches. In medium-sized operations located in the humid lowland forests of French Guiana, the effective use of electric fences resulted in a 98% reduction in losses (Table 1). In one medium-sized ranch (2994 ha) in the Chaco and Chiquitania ecotone in Bolivia, an increased proportion of buffalo, combined with electric fence, night corrals, and enclosures with electric lights, strategic placement of nutrition supplements, and a ban on hunting, resulted in substantial reductions in losses to jaguar attacks, with the ranch owner attributing a high proportion of the success to the use of buffalo. Electric fences in the Atlantic Forest of Misiones, Argentina, prevented 100% of jaguar depredations in three medium and large cattle properties (200 to 2500 head of cattle, 60–2500 ha) for over a decade [61].

Of 194 livestock operations with metrics on ADS effectiveness, only 24 provided sufficient data to assess the cost-effectiveness of the implemented interventions. Evaluations of the cost-effectiveness of the ADS on these 24 livestock operations across three countries (Paraguay, Brazil, and Colombia) found that benefits in livestock losses averted exceeded the investments required in 88% of the cases (Table 2). In 21% of the livestock operations (five of 24 total), benefits exceeded investment by more than ten times (11.6–22.1 times). In 17% (four of 24 total) of operations, benefits exceeded investments by 3.1 to 10.0 times. The highest proportion (50%) of operations experienced benefits between 1.0 and 3.0 times the investments. In 12% of operations, costs exceeded benefits, which was attributed to failing to apply the ADS consistently, with resulting abnormal losses skewing the results. An additional (non-measurable) benefit is the owners’/community’s ease of mind, knowing that their livestock is protected.

## 4. Discussion

ADS applications vary in complexity and require investment (see Table 3). In areas with challenging access, where roads and electricity are unavailable, the simplest and most effective strategies are enhancing animal husbandry, erecting fences, building night corrals, and maintaining the natural prey population (rows A, B, and C in Table 3). Introducing simple and affordable improvements in livestock management can increase productivity and reduce the chances of jaguar and puma attacks (refer to Table 1 and Table 3); evidence from Nicaragua supports this (discussed below). Efforts to improve livestock management and education at 43 farms, accessible only after two days of river travel, resulted in zero losses to jaguars during the project [60]. This level of simplicity may be suitable—possibly the only feasible choice—for more secluded areas like the Mamirauá Sustainable Development Reserve in the west-central Amazon (S2), as well as parts of the Upper Amazon and remote regions of the Guianan Shield. However, a collaborative process with local communities is essential to align interventions with their practices and priorities. Incorporating community perceptions and attitudes toward jaguars enhances the relevance and sustainability of coexistence strategies while fostering local stewardship. A participatory approach with technical experts and livestock producers working together will address logistical constraints while creating solutions that resonate with community needs and expectations. While improved livestock management can minimize losses, the best results typically arise from integrating various forms of ADS at multiple levels.

We noted that participating livestock operations that reported fewer losses implemented multiple ADS concurrently. Enhanced livestock management became a central theme, with intensity variations linked to available resources and local expertise. Furthermore, although veterinary care was quite advanced in numerous operations, some still required its assistance. The overall standard of quality in animal husbandry improved through specific ADS. The broader aspect of ADS, quality animal husbandry, was complemented by more specific elements; the combinations differed (see Table 1), but the results were consistently positive throughout (see Table 1 and Table 3). In the analysis of 24 livestock operations regarding benefit-to-cost ratios (refer to Table 2), 38% demonstrated that benefits were more than three times the costs, and 50% had benefits ranging from one to three times the costs. Together, these amount to 88% overall. In instances where costs surpassed benefits, the predominant reason for failure was the inconsistent implementation of ADS, leading to corresponding livestock losses.

Our findings, supported by Table 1 and Table 2, show that ADS are very effective, often exceeding their costs, sometimes by a significant margin, when evaluating financial benefits. De la Torre et al. (2021) [62] found that the benefit–cost ratios for 11 livestock operations in the Selva Lacandona of southern Mexico varied between 1.2 and 26.6. They noted that the benefits were more than double the costs in nine of the 11 operations (82%). The results from de la Torre et al. (2021) [62] were not included in Table 3 because their analysis considered a more comprehensive range of factors that needed to match the more focused scope of our analysis in this paper. A key takeaway from the de la Torre et al. (2021) [62] study is that ADS methods beyond improved husbandry, such as electric fences, could be successfully implemented even in a remote region. The authors attributed the project’s success to a thorough strategy that included outreach, education, robust community engagement, and animal care and livestock management improvements, complemented by persistent supervision and focused ADS techniques. The emphasis on a mixed strategy, advocated by a team working with small farms (where an average flock is 30 sheep and cattle herds average 90), was similarly evident in the largest operation in our sample. This operation, run by Instituto Homem Pantaneiro, managed 130,000 hectares with 30,000 livestock (see Table 1 and Table 2, and Supplementary Materials S2). Although the methods varied by location and operation, 93% of the participating sites indicated using multiple ADS within the same operations. The mixture of tools differed, which is appropriate for this discussion, and within the broader context of biological and carnivore conservation, where the choice of tools and their application intensity must be tailored to the specific characteristics of each site, owner, and situation.

Although tourism was not highlighted as a strategy against depredation—since it is not one—there are instances where it can significantly contribute to human–jaguar coexistence. Some livestock operations featured in this study incorporated tourism into their business models. This situation was observed during two operations in Brazil’s Pantanal, one in Bolivia’s Chiquitania/Chaco, two in Colombia’s Llanos, and one in Brazilian Varzea (seasonally flooded Amazon forest). Tortato et al. (2017) [63] discussed the benefits of jaguar-focused tourism in the Northern Pantanal, while studies have shown that tourism supports jaguar survival in some areas of Venezuela’s Llanos [64,65,66] when tourism emphasizes the importance of jaguars for ranches, communities, or regions, tolerance increases, coexistence improves, and jaguars become assets, instead of being liabilities [64]. In the Mamirauá Reserve in the Brazilian Amazon, community-based tourism and research activities have positively impacted human–jaguar relationships. Interviews conducted between 2020 and 2021 revealed that areas with these initiatives exhibited higher tolerance toward jaguars, more positive attitudes, and lower intentions to kill them [67].

Electric fences have become an effective tool widely used in various situations. While specific features—like length, number of strands, strand height, voltage, and power source—differ by location, 87% of sites that reported on ADS’s effectiveness (Table 1), used electric fences. These fences primarily target pastures and nighttime enclosures containing young animals alongside mother cows and calves, as these livestock areas and age classes are particularly vulnerable to predation [34,44]. A possible barrier to using electric fences is the limited expertise in rural areas and the associated maintenance costs. These issues can be addressed by involving specialized companies to train potential clients, along with support from technicians from government entities and NGOs. Electric fences offer extra advantages, including improved herd management via subdivided grazing areas, which allows for the creation of new paddocks and expanded rotation options. They can also provide energy supply possibilities for low-income households, enabling families to utilize the same energy sources that power the fences [59]. The implementation of electric lights yielded positive outcomes (see Table 1). However, experienced participants observed that jaguars are likely to adapt to the sensory disruptions caused by these lights and recognize that they do not present any genuine physical threat or discomfort. This contrasts sharply with an electric shock’s deterrent and repellent effects. If lights stay on for long durations, then their benefits can diminish, especially in areas with tourism-habituated jaguars. Therefore, it is advisable to utilize them for shorter spans, such as two months (sixty days), which corresponds to the peak vulnerability period for calves [34]. Observations and diagrams of electric fences can be found in Supplementary Materials S3.

We observed maintenance challenges related to electric fences in southern Mexico, French Guiana, Colombia, Venezuela, and Brazil, as discussed in Supplementary Materials S2. Generally, the solution hinges on the scale of electric fence installation, further justifying their use primarily during the most vulnerable phases of livestock. In this research, the most extensive pastures secured by electric fences measured around 100 ha. Cost comparisons between traditional and electric fences revealed some variations. In Colombia, traditional fences are priced at three times the cost of electric fences, while the differences in other areas are negligible.

In contrast, electric fences in French Guiana are more expensive than traditional options. Although there are some maintenance challenges, there is a general consensus that electric fences are effective and preferred deterrents. This strategy has the potential to significantly expand in various areas, as it safeguards at-risk age groups and livestock stages. Additionally, many maternity pastures and night enclosures are situated close to homes, providing an extra deterrent and enabling constant monitoring. This setup is recommended whenever possible.

The use of depredation-resistant livestock, such as criollo cattle and buffalo, has mainly yielded favorable results, particularly in Bolivia, Colombia, and Costa Rica. It is essential for field workers handling criollo cattle, and especially buffalo, to have time to adjust to new husbandry practices. Moreover, a market for these alternatives to traditional beef should be developed, or owners must be aware of related market trends. The low prices of buffalo meat are less of a concern because the performance of the meat carcass and the higher productivity of buffalo offset these lower market prices. While both criollo cattle and buffalo show remarkable resilience against depredation, there are situations in which neither species is entirely safe from jaguar attacks. In the Venezuelan silvopastoral systems, young buffalo were depredated nearly as often as cattle, mainly when the weaned buffaloes were managed without adult guard buffaloes. As herds mature and gain experience, the animals and their owners can adopt effective methods while viable product markets emerge, producing positive results. Buffaloes also present advantages due to their impressive milk production in tropical environments. Items like mozzarella cheese, “dulce de leche” (a dessert created with buffalo milk), and ricotta are greatly valued in local, national, and international markets throughout these countries.

A fundamental principle of ADS is minimizing the interaction between domestic animals and predators, aiming to “keep livestock away from forests and shared water sources.” This strategy includes suids (pigs), sheep and goats, bovids (cows and buffalo), and equids (horses) [16,52,59]. Experiences from Nicaragua highlight the irregular presence of shade trees, varying forage banks, and nitrogen-fixing living fences in silvopastoral systems. In contrast, agroforestry and silvopastoral practices in Venezuela focus on a system that enables cattle and buffalo to graze within forest plantations. Despite the advantages, these systems face unique challenges.

Nonetheless, using electric fences combined with lighting has significantly reduced livestock losses, even in these difficult situations (Table 1). A fundamental aspect of wildlife conservation is maintaining a robust natural prey population to offer jaguars alternatives to livestock [68]. Game species are intricately linked to jaguar prey [32]. Despite being crucial for reducing depredation [34], only 73% of our sites implemented explicit, partial, or total hunting bans on natural prey. Addressing this issue is vital for creating effective ADS systems despite the challenges.

There is frequently a division between special interests, such as biologists, who focus on forests, carbon capture, mitigating climate change, and biodiversity conservation, and those who do not necessarily see all livestock-rearing practices conflicting with these goals. This divide may arise from personal experiences or entrenched beliefs. People who have primarily lived in areas with mostly uniform forests, suffering significant damage from poorly managed and ever-expanding livestock operations, frequently view all ranching as detrimental to the environment. Conversely, individuals who have participated, resided, and worked within well-managed livestock systems that successfully maintain species diversity, forests, and watersheds understand that achieving a balance is challenging yet feasible, as demonstrated [15,69,70]. For example, in this study, forest cover made up roughly 46% of the land across nine ranches in Paraguay, ranging from 4000 to 45,000 ha, which supported between 1100 and 19,800 head of cattle. In the Llanos of Venezuela, Polisar et al. (2003) [34] noted that forests constituted 50% of the area studied, while in the three Pantanal ranches included in this research, at least 40% of the area consisted of protected riverine forests or forested savannas, with cattle reared chiefly on native grasses.

Approximately 53% of our sites feature specific provisions to conserve forests and habitats, encompassing mechanisms such as conservation agreements. This figure increases to nearly 67% when considering inferred habitat conservation related to loosely enforced state bans or proximity to nationally protected areas. This consideration requires careful balance. Pastures too close to forests, or small pastures within them, can lead to increased depredation rates, as jaguars prefer forest edges and avoid open spaces [33,49,71]. Enhancing the nutrition and health of livestock in a specific area is a way to reduce the need for pasture expansion and subsequent deforestation. Nonetheless, a clear link between higher livestock productivity per unit area—thanks to improved animal husbandry (including nutrition and health)—and a reduction in deforestation due to pasture expansion has been infrequently documented, even though it is feasible [59,60].

Protecting forests and habitats is essential for maintaining healthy prey populations and watersheds. It also provides jaguars with both land and water resources for their diet. The concept is that well-preserved forests and water bodies, devoid of cattle, form ideal habitats for prey, thereby supplying jaguars with the natural nutrition they need. Effectively managed and preserved forests can help to create distinct areas for jaguars away from livestock, reinforcing a previously discussed principle. To boost jaguar conservation, there is a pressing need for enhanced national-, local-, and ranch-level planning that prioritizes forest connectivity and pasture management. Many operations within this region are currently adopting these strategies. When appropriately executed, cattle ranching can contribute to forest conservation efforts [19]. Privately managed ranches and Indigenous lands, frequently well-protected, can sometimes surpass public protected areas in biodiversity conservation, as demonstrated by Devlin et al., 2023 [72].

To grasp the interactions between jaguars and livestock, it is crucial to observe both (1) jaguars to livestock and (2) livestock health on farms, ranches, and community operations. Throughout all study operations, jaguars were monitored along with pumas and sometimes feral dogs, utilizing a blend of camera traps and various signs, such as tracks, scrapes, feces, and kill traits. Moreover, one site implemented linear foot transects in addition to the previous methods. Livestock monitoring showed significant variation, which is anticipated due to the range of operations from around 5 hectares to 130,000 ha and between 30 and 30,000 animals. Yet, even if access and scale pose challenges, livestock monitoring is essential to the ADS suite [52,59]. In this study, nearly all operations conducted regular livestock inventories, although the frequency varied,ranging from weekly or biweekly to monthly or even once or twice a year. Dairy cattle are monitored daily during production. Holding inventories annually or bi-yearly cattle roundups may be insufficient for effectively tracking livestock conditions, including theft, losses, and gains, and supporting adaptive management strategies. One participating operation or group did not have a formal inventory system. Another country noted that only 2 out of 12 (17%) livestock operations performed regular inventories. This practice is an essential first step in monitoring livestock health, identifying the causes of mortality, and pinpointing areas within the operation where mortality rates are elevated, allowing for differentiation between causes such as predation that require attention. Interventions are most effectively evaluated when used alongside regular livestock inventories [73].

In developing this study, we asked about local involvement, the participation of farms and ranches, and methods to expand efforts to affect broader regions of jaguar habitat. We recognize that ADS initiatives are underway and are being assessed in almost all countries where jaguars are found, yet the percentage of land they cover remains likely relatively small. Local project engagement varied by scale. Some authors in this paper focused directly on ranches (Bolivia 1, Brazil 3, Venezuela 1). Other contributors, however, detailed significant involvement at both the local and farm levels, which includes (1) providing direct technical assistance; (2) organizing capacity-building workshops; (3) conducting ongoing communications and evaluations; (4) ensuring timely responses and actions when needed; (5) fostering strong ties with local communities to guarantee that jaguar conservation generates broader local benefits; and (6) encouraging jaguar-focused tourism when feasible, yielding specific local benefits. Practical approaches and cost-efficient interventions at the local level have proven to be the most compelling “advertisement”. In this respect, Costa Rica developed an exemplary national model of an ADS implementation program, The UACFel initiative, between a local NGO and government organizations, as described by Corrales et al. 2016a,b [74,75].

Building and maintaining relationships are crucial for success at every level. Projects that utilize years of collaboration can significantly benefit from these established connections. New initiatives should develop these relationships; nothing strengthens them more than reliable execution that meets commitments. Many participants noted that project performance affected farmers’ and ranchers’ perceptions of predators like jaguars. Improvements in livestock management, resulting in increased productivity, also led to better acceptance and should be integrated into any ADS projects to be carried out.

## 5. Conclusions

The best approach to improve the uptake of ADS is to showcase proven efficacy. We offer compelling evidence of the effectiveness of interventions across multiple operations and biomes, along with cost-effectiveness data for select interventions. While our summary includes specific examples spanning sixty degrees of latitude, we emphasize the importance of monitoring all ADS interventions to assess their efficiency and cost-effectiveness. Disseminating these findings will encourage broader engagement and overall uptake. We recommend active collaboration with cattlemen’s associations, publishing articles in local and national newspapers and online platforms, speaking at scientific and agricultural conferences, and organizing meetings with government agency staff and elected officials. While accurate information is crucial, it must be communicated effectively to decision-makers at every level—from local farms to the national stage—to drive change. Delivering the message in venues where it will reach the desired audience is crucial, be it a community center or a government office.

Challenges to implementation and evaluations, particularly in remote regions, may include (1) the lack of dependable channels for citizens to report issues and communicate needs to authorities or NGOs; (2) inadequate routine livestock inventories; (3) the absence of standardized reporting formats, or inconsistencies in timeliness, which can obstruct effective changes; (4) a deficiency in both government and cattlemen associations’ readiness, either fully or partially, to address these issues, alongside a lack of awareness about the tools available and inadequate organization to effectively promote and utilize these resources effectively for a broader impact. All these challenges can be overcome. We stress the importance of increasing the government awareness of and promoting these proven techniques. We urge governments within the jaguar range to enhance their understanding and embrace these efficient, cost-effective strategies.

The Jaguar 2030 Roadmap for the Americas [19] aims to improve jaguar conservation across the species’ range through the following four interconnected strategies: (1) coordinating efforts across the range to protect and link jaguars and their conservation landscapes; (2) implementing national jaguar conservation strategies; (3) significantly expanding the use of sustainable development tools in Jaguar Conservation Units (JCUs) and Corridors, including trans-boundary landscapes; and (4) achieving financial sustainability for jaguar conservation efforts. Pathway 3 emphasizes the importance of innovative strategies to reduce human–wildlife conflict, involving local communities and livestock operators through participatory methods, as detailed in this study. CITES Decision 19 has formalized the Jaguar Roadmap, requiring collaboration among Parties and the UN Convention on Migratory Species (CMS) to establish a Joint Program of Work, a Rangewide Monitoring Framework, and an Intergovernmental Platform [76]. Furthermore, jaguars are recognized as indicators of biodiversity, and preserving these apex predators is seen as an effective strategy for range states to fulfill their obligations under the UN Convention on Biological Diversity’s (CBD) Kunming-Montreal Global Biodiversity Framework [77], particularly in achieving Goal A, Target 4. The pragmatic human–jaguar coexistence tools analyzed here can contribute to the long-term persistence of jaguar populations. The benefits generated also aim for a larger scale. Jaguar conservation focuses on an apex predator and preserving the ecosystems it inhabits. Big picture jaguar conservation seeks a balance between human needs and the persistence of the natural world, stabilizing ecological life support systems that include watersheds, climate, and biological diversity. The tools that this study presents can contribute to that balance.

## Figures and Tables

**Table 1:** Efficacy impact of anti-depredation strategies.

Site/Biome, Country	Number of Operators	Size and Type of Livestock	Size Hectares	Types of Interventions	Efficacy/Reductions in Losses
Selva Lacandona, southern Mexico (lowland humid forest, with areas cleared for pastures)	Σ = 30 25 Ejidos 5 indigenous	Average cattle 90 Average sheep 50	Average 44 ha	Night enclosures for sheep, electric-fenced maternity pastures for cattle, no hunting in ejidos, improved husbandry.	97% reduction in losses of sheep via night enclosures 86% reduction in losses of vulnerable cattle classes (via electric fenced maternity pastures)
Chaco, Paraguay (low stature dry forest with areas cleared for pastures, areas abutting semi-deciduous Chiquitania forests, and the Pantanal watershed)	9 + private ranches	1400–18,000, six ranches over 10,000 head (cattle)	4000–45,60 ha, eight ranches over 10,000 ha	Electric lights, electric fences, bells on collars, strategic location water sources, donkeys as guard animals, no hunting, combinations.	Villalba et al. (2016) [58] included six ranches, including some under 1000 head, and others up to 38,627 ha and 17,500 head (Campo Grande), using electric lights for some, and combinations of six methods for others (electric lights, electric fences, husbandry, fence exclusion from forest edges, cleaner pastures, controlled birthing seasons, secure maternity pastures) yielded a 100% reduction in losses. More recently in Campo Grande, tests with donkeys yielded a 79% reduction in cattle losses.
Llanos, Colombia (seasonally dry and flooded savanna–forest mosaic)	22 ranches	101–500 head cattle	Average 1370 ha	Electric-fenced pasture for mother cows and calves and for horses, electric-fenced night enclosures for vulnerable (young classes), electrical fenced barriers excluding cattle from forest and rivers/gallery, active in-person vigilance, strategic placement of pastures (close to house), strategic placement of water sources, Creole Sanmartinero cattle 1 bull/50 Cebu cows, partial ban on hunting of prey and on deforestation.	Fourteen ranches (64%) with a 100% reduction in losses, six (27%) with greater than or equal to 75% reduction in losses, one with a 67% reduction, and one with only 43% reduction. The latter two due to weak and inconsistent compliance with the anti-depredation techniques. Initially, Sanmartinero were vulnerable to depredation but over time became accustomed to ranch with zero losses.
Tortuguero and Maquenque, Costa Rica (lowland humid forests with areas cleared for pastures)	13 ranches	520	67 ha	Electric fence on all ranches, and buffalo on four of them.	Electric fences eliminated (reduced by 100%) depredation on 12 (92%) of the ranches. One loss on one ranch, which is a 75% reduction from collective average losses annually.
Yungas, Argentina (Andean forests with lower and mid elevations cleared for pastures, some higher natural grasslands)	21 ranchers, community property, 10% edge of national park,	Σ 1300 cattle, some sheep, horses, mules, donkeys, pigs	Σ 80,000 ha in a nearly free-ranging system	Tests with 650 head cattle. Built fences for 16 pastures, four community pastures (12–15 ha), and 12 family pastures (~6 ha each). Used these for mothers with young, and animals under 3 years in age. The four larger community pastures were encircled with electric fence. Also, electric lights, bells, odorous collars, (judged highly effective), and combinations were used.	Electric fences yielded zero depredation (100% reduction in losses). Pastures with electric lights and near houses also had zero depredation, a 100% reduction in losses. Enclosure in potreros (with electric fence, without electric fence, with electric lights) eliminated losses. Losses were reduced but not eliminated by odorous collars or collars with lights and bells. Overall, losses were reduced by >50% by the pooled range of anti-depredation techniques. Simply stopping free-ranging reduced losses.
Sonoran (desert scrub), Sierra Madre Occidental, Northern Mexico	1 ranch	400 head cattle	7000 ha	Releasing and increasing natural prey (collared peccaries), ban on hunting.	A 50% reduction in depredation losses to jaguars.
Bosawas Biosphere Reserve, Nicaragua (lowland humid forest with areas cleared for pastures)	43 operators in communal titled indigenous territories	80% 1–25 head cattle, 20% 26–100 head cattle, 2017–2019: 80–92% owned 1–10 pigs, 2017–2019: 8–20% owned 11–15 pigs	All under 100 ha, majority 2017–2019: 66–83% < 49.9 ha pastures	Improved husbandry and livestock control via improved nutrition (improved pastures, silvopastoral systems, protein banks), improved veterinarian care, better contained livestock excluded from forest (standard fences and live fences), kept closer to dwellings, enhanced vigilance, complete ban on hunting of some game species, partial ban on hunting of others, complete ban on deforestation.	In this rustic setting (two days from nearest roads) reduction in livestock losses through improved livestock management was complete with a 100% reduction in losses.
Chiquitania-Chaco, Bolivia (savanna–forest mosaic, small amount cleared forest)	1	300 buffalo, 50 Cebu cattle, 25 pigs, 20 horses, 20 sheep, 5 mules	2994 ha	Increased proportion of buffalo to cattle, electric fence, night corrals/enclosures, with electric lights, strategic placement of nutritional supplements, complete ban on hunting, ban on deforestation, nature-based tourism.	Reduced losses since introduction of buffalo, less than 5/year, last year 1 horse and possibly 2 buffalo calves. Losses reduced, owner primarily attributes that to buffalo.
French Guiana coastline, extension of lowland humid Amazon Forest (small clearings for pasture)	4	80, 100, 250, 300 head of cattle	Average ~100 ha	Electric fences in 1 ha enclosures used for mothers and calves, or calves only. These constitute electrically fenced night enclosures, set a minimum of 100 m from forest.	Electric fences are potent deterrents, with a 98% reduction in losses where effectively deployed. This team had experimented some with electric lights and found their impact to be temporary/short term and less effective with jaguars already accustomed to taking livestock.
Belize, multiple sites (lowland humid forest, small clearings for pasture)	11	Sheep and pigs (8,8, and 20 head), remainder were cattle 9–300 head	From less than 1 ha to 120 ha	Electric lights (7 properties), fencing (1 property), night enclosure (1 property) ultra-sonic deterrent (1 property), cowbells (3 properties).	Cow bells alone (2 cases) resulted in no further attacks, night enclosure with cow bells (1 case) resulted in no further attacks; in 6/7 cases, lights were judged effective, and the one that was not needed more fencing, though also in 2/7 there was no clear evidence that jaguars still frequent the area, with the same issue for the sonic deterrent (judged effective, but continued presence jaguars not confirmed). Overall, enclosures combined with lights or bells judged effective.
Llanos. Venezuela (seasonally flooded savanna–forest mosaic, this project part of a silvo-pastoral agroforestry (emphasis forestry) system	1	1200 head cattle 1200 milking buffalo	13,897 ha	Emphasis on true silvopastoral system (livestock grazing in forest) in this system makes the complete separation of livestock and forest challenging. Anti-depredation strategies include portable electrified sheep-type night enclosures of ~1 ha, for high % of cattle emphasis on mothers and calves, and for 30% of buffalo, young (weaned) males and females of 1–2 years; tested lights in combination with electric fence night confinement; electric fence pastures of 5–10 ha, emphasis on mothers and calves, and strategically placed near guards; day and night active vigilance horseback and motorcycles; complete ban on hunting; complete ban on deforestation (other than planned extractive forestry); overall, tighter control of livestock.	Increasing livestock losses in 2017–2021 stimulated the initiation of anti-depredation strategies in this site. In 2022, installed electric fence maternity pastures, vigilance, night confinement cattle, and installation of lights decreased losses from 2021 of 8.7% cattle, 17.4% buffalo. In 2023, changes included cattle confinement in sheepfold electric fence with lights, night corrals, only daytime vigilance, and, for buffalo, night confinement in small pastures, 30% buffalo females, 1 year old males, with night and day vigilance. Cattle losses decreased by 5.35% and buffalo by 19.38% from 2022. Overall, there was a decrease of 13.52% in cattle losses, and 36.78% in buffalo losses from 2021, when the year with the highest levels of predation had been achieved.
Iguacu, Brazil, Atlantic Forest	32 farms and ranches with a variety of livestock, including cattle, horses, donkeys, sheep, pigs,	2 head to >1400 head –mostly cattle, but also some pigs, horses, donkeys, and sheep	2–1936 ha	Beef cattle: Electric lights, cow bells, Turere collars (lights on collars). Dairy cattle: Turere collars, electric lights, electric fence, one night enclosure, improvements in livestock management techniques (such as nutrition, sanitary, carcass disposal).	Out of 32 ranches—recurrence of depredation on only 9, (72% had a complete cessation of depredation). One of the nine owners did not follow guidance. In 8/9, the jaguar already “habituated” accustomed to invade and take, so methods did not work as well.
Pantanal, Brazil, seasonally flooded savanna–forest mosaic (Instituto Homem Pantaneiro)	1	30,000 head cattle, 200 head horses	130,000 ha	Electrified night enclosures (mothers and calves), electric lights in pastures (mothers and calves), bells on cows, fenced off forested areas, complete ban on hunting, forested corridors maintained for wildlife.	Depredation losses in areas without electric fences 5.92 to 22.8 times greater than the areas with APS implemented/losses are higher in brushy overgrown pastures.
Pantanal, Brazil, seasonally flooded savanna–forest mosaic (Fazenda Jofre Velho)	1	~100 head cattle/year (varies slightly), 25–30 buffalo, 20–25 pigs, 15–20 horses	9275 ha	Electrified night enclosures = primary current method, entire small cattle herd. Pantaneiro (criollo) bull’s offspring had marketing challenges. Buffalo breed (Murrah). Pantaneiro and buffalo deterred attacks, but final method is electrified night enclosures. Pigs are tightly enclosed, fed kitchen scraps. Complete ranch-wide ban on hunting. Ban on deforestation. Ecotourism.	Current and sustainable methods = electrified night enclosures for cattle, and for pigs, tight control/enclosures. Mortality rate by jaguars for cattle across four years = 2.7%. Counterfactual is average for area is ~10%. Estimated 50–67% reduction in losses to jaguar. Tight, enclosed management of pigs means losses to jaguar = not an issue.
Pantanal, Brazil, seasonally flooded savanna–forest mosaic (Fazenda Ipiranga)	1	2500 head cattle, 220 horses	7200 ha	Electrified night enclosures, used (1) immediately pre-birth; (2) first two weeks of calves’ life; (3) between 5 pm and 5 am; otherwise, mother and calf are in pastures. This is a time-limited application of electrified night enclosures, combined with a ban on hunting and deforestation, and with a successful ecotourism business.	ADS reduced losses to jaguars 3/15–3/8 rate, or 20–37.5% what they were prior, indicating reductions in losses to jaguars of 63.5–80%, in the specific pastures and periods when the techniques were applied. Outside of these spatially and temporally limited bounds, additional losses occurred. However, impressed by success where deployed, the ranch owner has plans to expand deployments.
Argentina Atlantic Forest	3 cattle farms	200–2500 cattle	60–2500 ha	Electrified fences with solar panels.	A 100% reduction in depredation in 12, 8, and 6 years of continuous implementation.

**Table 2:** Benefit–cost analysis across three countries and biomes.

Country	Site Name and Source	Anti-Depredation Strategy (APS) Intervention Technique Applied	APS Investment USD	Losses Avoided USD	**BENEFIT/COST RATIO ^2^**
Brazil Northern Pantanal	Jofre Velho (This paper)	Electrically fenced night enclosure, including milking buffaloes and first three years with Pantaneiro bulls; prohibition of hunting and deforestation	2680	4140	1.63 ^1a^
Brazil Northern Pantanal	Ipiranga (This paper)	Electrically fenced maternity corral; prohibition of hunting and deforestation	4000	10,000	2.5 ^1b^
Colombia	Casanare Llanos ^3^	Creole cattle (2 ranches # 1 and 9)	Ranch 1 = 2257 Ranch 9 = 7147 Average = 4702	Ranch 1 = 26,080 Ranch 9 = 10,2500 Average = 64,290	Ranch 1 = 11.55 Ranch 9 = 14.34 BEN.COST RATIO = 13.67
Colombia	Casanare Llanos ^3^	Electrically fenced weaning paddocks (2 ranches, #2 and 10)	Ranch 2 = 1728 Ranch 10 = 1063 Average = 1395	Ranch 2 = 4600 Ranch 10 = 2100 Average = 3350	Ranch 2 = 2.66 Ranch 10 = 1.97 BEN.COST RATIO = 2.40
Colombia	Casanare Llanos ^3^	Electrically fences maternity paddocks (6 ranches, # 3, 4, 5, 7, 8, and 16)	Ranch 3 = 1328 Ranch 4 = 133 *Ranch 5 = 4518* *Ranch 7 = 1927* Ranch 8 = 199 Ranch 16 = 3854 Average = 1993	Ranch 3 = 3000 Ranch 4 = 2838 *Ranch 5 = 1900* *Ranch 7 = 1500* Ranch 8 = 4400 Ranch 16 = 1767 Average = 2567	Ranch 3 = 2.25 Ranch 4 = 21.33 *Ranch 5 = 0.00* *Ranch 7 = 0.00* Ranch 8 = 22.11 Ranch 16 = 0.45 BEN. COST RATIO = 1.29
Colombia	Casanare Llanos ^3^	Electrically fenced night enclosures (4 ranches # 6, 12, 14, and 15)	Ranch 6 = 67 Ranch 12 = 133 Ranch 14 = 266 Ranch 15 = 366 Average = 832	Ranch 6 = 134 Ranch 12 = 480 Ranch 14 = 1280 Ranch 15 = 2320 Average = 4214	Ranch 6 = 2.00 Ranch 12 = 3.6 Ranch 14 = 4.81 Ranch 15 = 6.33 BEN.COST RATIO = 5.06
Colombia	Casanare Llanos ^3^	Electrically fenced riverine forest barriers (2 ranches #11 and 13)	Ranch 11 = 5290 Ranch 13 = 2116 Average = 3703	Ranch 11 = 11,800 Ranch 13 = 4200 Average = 8000	Ranch 11 = 2.23 Ranch 13 = 1.98 BEN.COST RATIO = 2.16
All Colombia	Casanare Llanos ^3^	All 5 anti-depredation strategies were applied, and no hunting and no deforestation in all 16 ranches.	16 Ranches Average = 2024	16 Ranches Average = 10,681	16 RANCHES BEN. COST RATIO = 5.28
Paraguay	Paraguay North-Central Chaco ^4, 5^	LED Lights (3 Ranches #1, 2, and 3) No-wild-prey hunting policies	Ranch 1 = 800 Ranch 2 = 400 Ranch 3 = 400 Average = 533	Ranch 1 = 4700 Ranch 2 = 1200 Ranch 3 = 7020 Average = 4307	Ranch 1 = 5.87 Ranch 2 = 3.00 Ranch 3 = 17.55 BEN.COST RATIO = 8.08
Paraguay	Paraguay North-Central Chaco ^4, 5^	LED LIGHTS + wlectrical fences, clean areas around pasture paddocks, clean night-sleeping paddocks, breeding seasons (3 Ranches # 4, 5, and 6). No-wild-prey hunting policies.	Ranch 4 = 1630 Ranch 5 = 1630 Ranch 6 = 1630 Average = 1630	Ranch 4 = 2100 Ranch 5 = 2520 Ranch 6 = 4200 Average = 2940	Ranch 4 = 1.29 Ranch 5 = 1.55 Ranch 6 = 2.58 BEN.COST RATIO = 1.80
All Paraguay	Paraguay North-Central Chaco ^4, 5^	All anti-predation strategies applied, and no hunting in all 6 ranches	6 Ranches Average = 1082	6 Ranches Average = 3623	6 Ranches BEN. COST RATIO = 3.35
All 24 ranches	Pantanal, Llanos, Chaco—Brazil, Colombia, Paraguay	All ranches and anti-depredation techniques pooled, calculating the 24-ranch average benefit–cost ratio.	45.562/24 = 1898	206.779/24 = 8616	24 ranches average BEN. COST RATIOS = 4.54

^1a^ Jofre Velho calculations: A four-year total inventory of 409 Animals. Mortality from other causes was 9.5%, and mortality caused by jaguars was 2.7%. Usual mortality by jaguars is estimated from data from neighboring Fazenda São Bento with similar environmental conditions at 5%, equating to 20.5 total animals, based on the inventory of the 409 animals that would have been lost without APS. With the application of the APS, the total losses from predation were only 11 animals during the four years, or 2.7%. A difference of 9.5 animals @ 2.300 R$ = USD 460 each 9.5 × USD 460 = USD 4.370 losses avoided. Price of building and maintenance of the electrified night enclosure = USD 2.680. Benefit–cost ratio index = 4.370/2680 = 1.63. ^1b^ Ipiranga calculations: The average number of animals lost due to predation during the 2021/2022 and 2022/2023 cycles, before the installation, was 18 animals. Installation cost: USD 4000. Average value per animal: USD 400. Losses avoided in the 1st year: 8 animals. Losses avoided in the 2nd year: 17 animals. Value of losses avoided in the 1st year: 8 animals × 400 USD = 3200 USD. Value of losses avoided in the 2nd year: 17 animals × 400 USD = 6800 USD. The total value of losses avoided, 3200 USD + 6800 USD = 10,000 USD. Overall cost–benefit ratio:10,000/4000 = 2.5. ^2^ Decimals were rounded up (e.g., Ranch 3 Casanare 3000/1328 = 2.259 = 2.26). We averaged the 16 Ranches of Colombia and the 6 Ranches of the Paraguayan Chaco at the end of each country for APS invested and losses avoided and then divided those averages for a round figure of the benefit–cost ratio for each country. ^3^ Colombia Casanare llanos calculations come from the following reference ([59] with two corrections in italics (*Except in ranches 5 and 7 the investments in APS exceeded losses averted (losses averted did not offset costs) so BENEFIT/COST RATIO is (less than) zero, and in italics*). ^4^ Paraguay calculations come from the following reference [58]. ^5^ Names of the six Paraguayan Chaco Ranches: Ranch 1 = Campo Grande; Ranch 2 = Kuarahy Reta; Ranch 3 = Los Ceibos; Ranch 4 = El Triángulo; Ranch 5 = María Belén; Ranch 6 = Isla Sola.

**Table 3:** Intervention types along the investment and complexity spectrum.

Predation Mitigation Method			Level of Complexity and/or Investment		
Strategy Classification		Options	Low	Medium	High
lA. Improved husbandry		Nutrition, health, reproduction	Improved pastures, agroforestry systems are linked to exclusion of livestock from forest, cadaver clean up protocols.	Basic veterinary care, involving training and materials, supplementary salt, balanced minerals, adjustment to fit carrying capacity, improved pastures.	Advanced veterinary care, immunizations, artificial insemination for tightly controlled reproductive seasons, no longer than 4 months of calving
B. Physical, emphasis on protecting the most vulnerable ages/stages of livestock, including (with cattle) calving areas and successive period in which mothers are with young calves	Fences and night corrals	Fenced pastures with paddocks, no free roaming livestock, exclusion from forest.	Live fences, barbed wire fences, other materials, whether unconventional local materials or purchased, night enclosures without electric fence or lights.	Electric fences of relatively small scale and lower levels of investments (e.g., small pastures and maternities, small night enclosures)	Electric fences that require higher levels of investment (e.g., larger scale paddocks and pastures and larger-scale electrified night enclosures). Depending on farm size, all or part of it.
		Corrals, night enclosures, maternity paddocks, paddocks for young age animals (just weaned).			
		Barriers between livestock and forest, riparian, or otherwise.			
	Electric lights	On premises		Lights enclosing pastures and paddocks, lights on night corrals, and night enclosures	Motion-sensor LED lights, with or without sensor alarms.
		On animals		Collars with intermittent LED lights (with/without bells)	
	Auditory deterrents	On premises	Fire-works, pyrotechnics, butane gas-powered cannon		
		On animals	Cowbells on reflective collars, with or without lights		
	Visual deterrents		Fladry on fences	Wind dancers or puppets	
C. Administration and Management			No hunting, no deforestation, no feral dogs, no dogs at all	Strategically located maternity pastures (clean and far away from forested areas), strategically located water and/or nutrition sources, water sources out of forested areas	Reintroduction of prey, jaguar and nature tourism, insurance and reward strategies for farmers and ranchers that comply with ADS, no hunting, and no deforestation, receive premium for lost animals
D. Defense	Human vigilance	Day, night, both	Vigilance walking or horseback	Vigilance using tractors, motorcycles or boats, fireworks	
	Guard animals	Depredation resistant species (buffalo) and breeds (criollo) races, resistant species, or trained guard animals (guardian dogs that sleep with the animals), and, in some cases, donkeys.	Strategic placement of mature experienced resistant ages and stages with more vulnerable ones, or donkeys	Criollo cattle, trained dogs, camelids	Buffalo
E. Chemical	Collars				Collars with chemical deterrents (olfactory and taste)
	Deterrents				Other methods by chemical deterrents are delivered

## Data Availability

None were deposited in an official repository. Data can be provided upon written consent directed to the corresponding author.

## Supplementary Materials

The following supporting information can be downloaded at https://www.mdpi.com/article/10.3390/ani15091247/s1: Structured Questionnaire-011152024, S2: Summarization 1st parts questionnaire 01122025, S3: Observations, diagrams, and comments on anti-depredation electric fences + lights 01122025 Final, S4: Broad acknowledgements Team x Team.
