# Supplementary material for: Sixty Degrees of Solutions: Field Techniques for Human–Jaguar Coexistence
Source: Animals (Basel). 2025 Apr 28;15(9):1247. doi: 10.3390/ani15091247 (PMC12071174; doi:10.3390/ani15091247)
Supplement: Supplementary file 1 [file animals-15-01247-s001.zip › S1. Structured questionnaire - 01152024.pdf]

Encuesta Estructurada / Questionnaire for paper

Title: Sixty degrees of solutions: field techniques for human-jaguar coexistence
Journal: Animals

Special Issue: Innovative Methods to Reduce Predation and Achieve Coexistence between Predators and Prey

Concept: The current range of the jaguar (*Panthera onca*) covers about 7,000,000km², approximately sixty degrees of latitude, and eighteen countries in the Western Hemisphere. Throughout this geographical breadth, jaguars represent an important component of native biological diversity and serve as an indicator of relatively intact ecosystems. The wide-ranging species often overlaps in space with humans, and conflict revolving around jaguar predation on livestock is a factor in jaguar mortality across this vast expanse. Technologies and practices have been developed to reduce the frequency of jaguar depredation across the entire spectrum of livestock operations and ecosystems, increasing tolerance and reducing retaliation. We present field-tested methods that work across the multiple biomes that constitute the jaguar range, providing recommendations for technologies tailored to the scale of operations and the ecological and cultural context in which they occur. These techniques are also effective in preventing or controlling depredations of livestock by other large carnivores, especially Pumas (along all the jaguar's distribution), and in some areas, Coyotes and Andean Bears.

Your completion of this questionnaire means that you give informed consent for the results of the questionnaire to be used in the publication. You understand that your participation is completely voluntary. You also know that you can withdraw at any moment without needing to provide a reason or incur any costs. Your participation and completion of this questionnaire is acknowledgement that you will be a co-author of this study and willingly agree to participate**.**

Al completar este cuestionario significa que usted da su consentimiento informado para que los resultados del cuestionario se utilicen en la publicación. Entiendes que tu participación es completamente voluntaria. También sabes que puedes retirarte en cualquier momento sin necesidad de dar ningún motivo ni incurrir en ningún coste. Su participación y finalización de este cuestionario es un reconocimiento de que será coautor de este estudio y que aceptará participar de buena gana.

Usar el espacio que necesita a contestar/llenar un formulario

Use the space that you need to fill out the information.

If you are part of a "group"/team e.g. Colombian llanos, Belizean lowlands, Argentine Yungas, perhaps also Venezuela (Cojedes etc.), Paraguay, Costa Rica, Sonoran Mexico, etc. – if part of a group – please work together and one person in group coordinate and submit the evaluation. Thanks!

Encuesta Structurada/Campos para datos: Siguiente

Por favor, nombre y titulo, institución e información de contacto del **Punto de Contacto** del equipo llenando el formulario? Representing which co-authors?
Please, name, title, institution, and contact information of the **POC (Point of Contact)** of the team/group participating? Representing which-co-authors?
Preferred email address if change is needed.

A. STUDY PARTICIPANTS AND STUDY AREA

Name(s) of individual or team?

Institution?

Country?

Biome / Ecosystem?

Physical location of site? Latitude and Longitude (Please remember to calibrate your GPS)

Inside a Jaguar Conservation Unit (JCU)?
https://www.internationaljaguarday.org/jaguar-conservation-roadmap

Or within a recognized, mapped Jaguar Corridor (per 2030 Jaguar Conservation Roadmap)
https://www.internationaljaguarday.org/jaguar-conservation-roadmap

Or outside of a JCU or Corridor, but within 30 km of either?

Or outside mapped, recognized JCUs and Corridors, yet with the presence of jaguars and human-jaguar conflict associated with livestock – in need of solutions?

What is the general widely recognized name of the area reporting upon?

The pattern of land ownership in the general study area/site surroundings?

- **Federal land?**
- **State land?**
- **Municipal land?**
- **Private?**
- **Community?**
- **Indigenous territories?**

IF ANY CATEGORY DOES NOT APPLY TO YOUR SITE / OPERATION, PLEASE TYPE IN NA = NOT APPLICABLE (TO YOUR AREA). NO PROBLEM. THANKS!

General categories will suffice when/where one team reports on ranches/farms of similar types and scales and methods tested. Please provide a number of farms (e.g., 39 in Nicaraguan Moskitia, 12 in Buffer Zone Maya Biosphere Reserve, Guatemala, 12 in S Mexico Selva Lacandona, five small and five large in Paraguayan Chaco, etc.)

If there are several widely varying sized ranches and farm types within the scope of your report – please divide by common/similar characteristics and submit separate reports – based upon their characteristics.

If depredation/anti-depredation techniques applied vary greatly among the farms, please submit separate reports for each <u>(broad)</u> category of operation/anti-depredation technique methods.

For example, if reporting on five ranches of 101-300 heads and 5 of over 5,000 heads, please submit separate reports for each size class. The same may apply to 5 ranches with anti-predation techniques type A & B and five ranches with anti-predation techniques C & D. Thanks!

If separate reports are necessary (as above) because within a reporting team, there are contrasting types of ranch/farm/operations or strongly contrasting scales, or the team deployed contrastingly different methods (within one reporting team), please submit separate reports for each (using <u>broad and general categories</u> of ranch/farm scale and biome for each category). Please do not "clump" or "pool" extremely variable-scale livestock operations (doing so may mask clear results), but please also not be "overly fine-grained."

Usar el espacio que necesita a contestar/llenar un formulario

Use the space that you need to fill out the information.

Parameters to consider follow.

B. SITE CHARACTERISTICS

Land ownership in/on specific site/s?

Public land? What type?

Private property?
Ejido?
Cooperative or Mennonite property?
Indigenous territory?

Ranch/farm size in ha?

> **If there are several to many operations within the operational unit, what is the range in size (in ha) and average size in ha?**

Size of operation in # of livestock?
For example: 0-19, 11-25, 26-50, 51-100, 101-500, 501-1,000, 1,001-3,000, 3,001-5,000, 5,001-10,000, >10,000?

Site distance (Euclidian?) to a community of over 2,500 people?

Site distance (Euclidian?) to a community of over 25,000 people?

Approximate distance (Euclidian?) to the nearest dirt road? Nearest paved road?

C. LIVESTOCK CHARACTERISTICS

Type of livestock? Approximate numbers in operation or in operations? If need to delineate several different farms/ranches, that is OK. If all are similar, then please provide the general range and averages.

Cattle
Buffalo
Pigs
Horses
Mules
Burros/Donkeys
Sheep
Goats

What is the purpose of cattle: Meat, dairy, double-propósito? Sales?

Races? In multiple, approximate numbers of each.

If criollos or buffaloes resist depredation, what is the size of the herd, solo, or mixed, and in what area (size of the paddock)?

Proportions of mixed herds by race.

D. PASTURES

Size of pastures in ha?
Ranges and averages if several/many pastures

Natural savanna or cleared former forest?

Introduced (exotic) pastures? E.G. *Brachiaria, Panicums*, other?

Fodder banks?

Are pastures surrounded by open areas (areas abiertas)?

Are pastures surrounded by forest?

Trees within pastures?
- **Over 40% coverage?**
- **20-40% coverage?**
- **Less than 20% coverage?**
- **None, or nearly so?**

IF A CATEGORY DOES NOT APPLY TO YOUR SITE / OPERATION PLEASE TYPE IN NA = NOT APPLICABLE (TO YOUR AREA). THANKS!

E. MANAGEMENT

Same pastures year-round?
Or
Rotate?

Move livestock seasonally?
An approximation of number of paddocks if available.

<u>Extensive?</u>
EXTENSIVE CATTLE PASTURES DEFINITION: Unfenced areas with native grasses / pastures, grazed all the year. In some areas like flooded savannas, grazed depending on season, flooded or dry season. Large extensions in which cattle roam freely, with a low level of management. Use of only salt (& sometimes minerals) as supplements (no use of feedstuffs or silage).

<u>Semi-intensive?</u>
Ranch / Farm fenced in paddocks in which the cattle is grazed. More use of introduced pastures, grazing rotation, more herd management e.g. breeding season, use of salt & minerals, use of feedstuffs (agriculture derived like cottonseed meal, maize-bran, soybean by-products, silage, fodder banks etc.), all year or during the dry season.

<u>Intensive?</u>
Livestock herds maintained in corrals or small pickets / paddocks, and intensively fed with hay, silage, recently cut fresh pasture and high use of feedstuff rations of agricultural by-products.

Regular inventories of livestock? Yes/no?

If yes, how frequently?

Nutritional supplements (salt/concentrates, protein additives, hay, silage, or protein banks such as *Leucaena*?

Sanitary programs/vaccinations? Yes/no?

Specify which vaccines are mandatory for your area if you know.

If yes to vaccines, which vaccines against which diseases and with what frequency?

Applied to what percent of livestock?

To what ages and stages?

Deworming programs/ endo- & ecto-parasites? Yes/no?

If yes, which ones and with what frequency?

Applied to what percent of livestock?

To what ages and stages?

Calf treatment after birth?

Artificial insemination, controlled reproductive season (and locations?)

Breeding Season (in the case of cattle, limiting the months in which the bulls get the cows pregnant to have a concentrated calving season in clean paddocks in which newborn calves get more intensive and better attention

F. PURPOSE OF LIVESTOCK

Production / sale of Seed- Bulls (Purebred Herds of higher genetics)

Steer Rearing (up to fattening age / weight)

Steer Fattening

Auto-consumption?

Sale at weaning – meat?

Sale as adults – meat?

Milk production?

Cheese production or other by-products (butter, yogurt, ice-cream, etc.)?

Both?

Other?

G. CORE ISSUES

1. Attacks

Frequency of depredation prior to anti-depredation techniques

Losses per year attributable to jaguar (and puma) attacks

Number per year? Age classes and sex of animals attacked (if possible)

Rate/proportion per year (as a percentage of total livestock with total herd as denominator and losses attributable to jaguar (and puma) as numerator)

When attacks occur –

What type of livestock?

What age/stage?

If known

What frequency of jaguar attacks?

What frequency of puma attacks?

Losses due to dogs, or other predators (e.g. Coyotes, Andean Bears, others?

2. How did you determine where to deploy what methods?

(please explain what factors and /or data considered on where to deploy anti-depredation techniques, and if the owner / manager participated in this decision, or simply accepted your advice)

3. Methods (tools to reduce depredation on livestock and to confirm presence of jaguars/wild carnivores)

In the next two sections, please consider what was the investment required to deploy the anti-depredation systems, what were the sources of support that enabled them, how sustainable that support may be, how sustainable the systems may be in the socio-economic/cultural setting in which the work took place.

Where possible – provide/insert an assessment of the investment, cost of the implementation (this can be useful to compare to savings achieved through techniques by reducing losses to depredation averted).

Please also insert some notes about associated training provided in the field.

State if the Predation mitigation method was effective. And if so, how?

Predator Mitigation Methods (PMM)

Night corrals/enclosures?

- **Type and stage of livestock contained?**
- **All? Mothers and calves? Calves? Other?**
- **Roofed – or not?**
- **Electrified – or not?**
- **More than one so they can be rotated in case of muddy conditions?**
- **Construction materials (tires, local wood, chicken fence etc.)**

Electric lights

- **Type?**
- **Size of pasture/corral / enclosure**
- **Type and age of livestock**
  - **All?**
  - **Mothers and calves?**
  - **Calves?**
  - **Other?**

Electric fences

- **Size of enclosure?**
- **Electricity source?**
- **Number of wires?**
- **Distance (cm) separating wires from the ground up.**
- **Which wires had electricity**
- **Voltage?**

All livestock?
Mothers and calves?
Calves?
Other?

Electrically fenced night enclosures (EF)
EF maternity paddocks?
EF "special paddocks" (weaned animals' young heifers)?
EF Riverine/Forest Barriers?
EF surrounding the whole farm (used in smaller properties)

Regular fencing, set back from the forest?
Distance to the forest?

Active in-person vigilance?
If so,
Type?
How?
Regular maintenance, weekly, bi-weekly, monthly?

Bells on cows?

Combination bells/lights in collars?

Strategic placement of maternity pastures?
If so, please comment

Strategical placement of nutritional supplements/rich food sources to influence livestock location?

Strategical placement of water sources to influence livestock (and /or wild prey) placement / distribution?

Fencing off forested areas and water sources in the forest?

Use of water buffalo?

Use of criollo breed cattle?
As criollo herds?
Mixed with Cebu-commercial herds?

If mixed – buffalo with cattle, criollo with other cattle, in what proportions?
Which breed / breeds, are used ?

Guard animals?
Donkeys?
# donkey / # livestock?
Dogs?
# dogs / # livestock?

Fladry / Flagging?

Complete or partial ban on hunting of prey? If so, please describe.

Complete or partial ban on deforestation? Maintenance of forested areas and corridors. If so, please describe.

Halting free-roaming (establishing fenced pastures/paddocks where previously there were none and livestock wandered freely)

Combination "improved husbandry" (nutrition/health) and anti-depredation techniques?

Please describe

Where some insurance or compensations are deployed, please describe them

Other?

Methods to confirm jaguar presence? Yes/no

Pre-study?

During study?

Post study?

Camera traps?

Sign (tracks, scrapes, typical carcasses, etc.)?

Estimate of time spent in outreach and training to generate trust, evaluate needs, generate solutions, help install and assess the techniques (evaluation and training and monitoring)? Costs?

4. **Results** (where possible assess cost-effectiveness, investments made in anti-depredation techniques and money saved in losses averted)

How did you know there were predation problems in facility?

Time deployed (please describe the time interval down to months that every anti-predation technique was deployed. This could be specific techniques (such as night corrals, e-fences, e-lights, etc.), could be type of livestock, could be pasture management, could be cattle movement, could be herd composition, etc.).

Each strategy – was deployed at what type of place between date XX-YY-ZZZZ and date XX-YY-ZZZZ, or at least, duration?

Some sense of relative effort, temporally, spatially – is useful to gauge scale of impact / relative impacts across / among study sites -

AND for similar reasons, OPTIMALLY also provide denominators, 1) of herd size in treated area, and 2) of total farm area and total livestock in farm involved with/in the "treatment" (technique).

Inventories are an important way to assess losses, impacts, successes.

Aim for assessments of pre-and-post losses, efficacy, of impact, cost-effectiveness.

Where possible provide PRE AND POST, before (technique applied) and after (technique applied) comparisons (including levels and % of mortality), or, alternatively, inside and outside the technique.

From each study site, for each method, please describe:

> *Time involved in / duration of deployment / evaluation?*
>
> *Scale (e.g., one pasture, several pastures)?*
>
> *Overall scale in this pilot (ha, head (where possible)?*
>
> *Scale relative to entire ranch / farm, relative to entire herd?*
>
> *Methods for monitoring losses, losses averted?*
>
> *What specific calculations were used?*
>
> *Methods and design to monitor jaguars (and other carnivores) in immediate project area?*

Parameters should include

Final summary of techniques deployed in each project area, at what scale, size of pastures,

numbers of pastures,

number of ha,

number of head involved.

If combined with improvements to nutrition and health, describe and document (succinct summary) those as well.

Reductions in predation, by livestock type

> *by numbers,*
>
> *by stage,*

by technique,

by combination of techniques

The above should have a denominator of herd size in the treated area so we can assess A/Z quantity (proportion) or livestock "saved."

Also, treated pastures are what % of total pastures?
Livestock involved with intervention are what % of total livestock?
In other words, denominators to facilitate interpretations of scale of impact.

Here, can be "fine-grained":
Livestock race, stage, age of livestock at risk,
and
race, age, stage of livestock "saved" by intervention

In each system, which intervention was the most successful, impactful?

Losses averted (same as above, said differently)?

Observations on maintenance requirements of systems, annual input, annual investments needed to keep "running"? IMPORTANT.

Comparison between conventional and electric fence, building costs and maintenance costs. VERY IMPORTANT.

Investments in each technique, and where several, the sum?

Estimates of cost-effectiveness (investment compared to immediate value of losses averted (sum livestock prices at specific age/stage of loss averted, as well as the same calculated if reach maturity)?

Examples of three ways to evaluate value of livestock (losses averted)

Villalba et al. 2016 (Paraguay) Unit of savings (for comparison was a ternero promedio (an average calf) with cost varying between small and large operations (probably quality and market based). Authors mentioned higher costs when genetic investments had been made in calves, but worked with two units, cost of average calf on small ranches ($200-300), and cost of average calf on large ranches ($350-500).

De la Torre et al. 2021. (S Mexico). Unit was average price of cattle ($540) or sheep ($78.75).

Valderrama-Vasquez et al. 2023. Cattle (stage?) $500, horse $400, pig $67, sheep $40. Not much detail, but useful range (based on real livestock prices in Colombia, in other countries they can be higher or lower, but we could use them as standard).

For your country/area – your estimated costs of standard calf, cow, horse, pig, sheep (as relevant to the operations in your study area). Please provide the numbers and types

and stages of livestock (whose loss was averted), as well as your estimated value (per unit, and total).

We may need to assign uniform average prices across all sites/countries.

5. DISCUSSION AND CONCLUSIONS MATERIAL (FROM YOUR AREA)

Source of support for the interventions (an important parameter to gauge long-term sustainability and feasibility of uptake in local, regional, national context)?

Government, grants, foundations, private

Duration of support for pilots

Opportunities to "scale up" using that support. Comms with Cattlemen Associations

Gap existing if want to scale up? Of what sort?

Additional observations, data, jaguars, jaguar attacks, before, after?

Your assessment about which techniques / methods seemed most sustainable in your area?

Did your interventions contribute to reduced deforestation, or forest recovery, and reduced poaching? and if so, how?

Please feel free to share additional observations and comments

Within your project area, of tools deployed, which seemed to have the most impact?

What "mixtures" contributed to overall success?
Did the methods deployed contribute to generalized higher farm or ranch productivity? Or not? If they did, how so?

If study has a general before and after socio-economic study, what were some of the most notable results?

Which intervention, PMM or otherwise, seemed highest potential to scale up, increase area of impact 2-5x?

Existing mechanisms to perpetuate and scale this up?

Gaps to address to accomplish a scale-up of 2-5 times?

Any gaps as far as government policies and practices to address to improve management?
