# Supplementary material for: Sixty Degrees of Solutions: Field Techniques for Human–Jaguar Coexistence
Source: Animals (Basel). 2025 Apr 28;15(9):1247. doi: 10.3390/ani15091247 (PMC12071174; doi:10.3390/ani15091247)
Supplement: Supplementary file 1 [file animals-15-01247-s001.zip › S3. Observations, diagrams, and comments on anti-depredation electric fences & lights 01122025FINAL.pdf]

COMMENTS AND DIAGRAMS OF ANTI-PREDATION ELECTRIC FENCES (APEF) USED TO REDUCE JAGUAR/LIVESTOCK CONFLICTS, WITH ADDITIONAL COMMENTS ON ELECTRIC LIGHTS.

Rafael Hoogesteijn and Almira Hoogesteijn

INTRODUCTION

Anti-predation electric fences (APEF) are designed in a way that livestock cannot leave the enclosure, and felines cannot enter the corrals/pastures where the livestock is, surrounded by the APEF. They are built in four different ways:
A) Night Enclosures: Small electrically fenced enclosures such as paddocks or corrals, in which the livestock herds are herded and sheltered only at night. Applicable on farms of any size and management.
B) Maternity Paddocks: Small or varied sized pastures / paddocks that are electrically fenced-in, generally used as maternity wards, where the most vulnerable species / age categories are placed: Sheep, goats, cows or mares just before giving birth or with their young at their side; also enclosing weaned animals.
C) Fencing the entire perimeter of the Property: A third option is to completely fence the entire perimeter of the property with these electric fences (on small and medium-sized farms), and all animals are protected at all times.
D) Riparian Forest Corridors: A fourth option is to create electric fence corridors along the watercourses with riparian (or riverine / gallery) forests that cross the property, with fence overhangs protecting the sides of the pasture. In this way, the felines roam and hunt along these watercourses with forested margins, without entering the pastures and the cattle do not go out to drink from these water sources or to browse in the forests, avoiding contact with the felines.

1) APEF STRUCTURE:

The structure of these fences can be observed in Figures 1-3, which show the diagrams of these effective electric fences that include a solar cell that captures solar energy and, through a regulator, feeds the battery, which in turn feeds an electric impeller, which sends the intermittent pulses of electricity to the positively electrified wire strands. It also has a protection device (lightning rod or arrester) against the destructive effects of lightning and thunderstorms. These fences have been successfully implemented in Colombia, Costa Rica, Panama, Brazil, Argentina, Mexico and Paraguay. APEF can also be placed laterally to existing conventional fences. With the conventional fence (plain or barbed wire) properly arranged and tensioned, two or three lines of electric wires are placed with insulators, on the outside of the conventional fence (to repel predators), at the distances from the ground specified in the diagrams, and the ground and the conventional fence strands are used as negative grounds.
The most important area to be electrically protected in any breeding/rearing livestock property is the maternity area or the delivery/maternity paddocks, where in the case of cattle and horses, newly calved cows (or mares) and their calves (or foals) must remain protected until at least three months of age, preferably until weaning (7-8 months of age).

2A) INITIAL APEF USED IN VENEZUELA, DIAGRAM 1:

In Diagram 1, we observe the basic design of the APEF used by Scognamillo *et al.*, 2002; the first APEF used in the field in a practical and successful way to control predation by Jaguars and Pumas in maternity paddocks at Hato Piñero in the Llanos of the Cojedes State, Venezuela (in joint work with the University of Florida in Gainesville).

DIAGRAM 1: Simplified diagram of the first electric fences used successfully in Latin America (Llanos of Cojedes, Venezuela) to repel attacks by predatory felines, placed on

the outside of a maternity paddock (Modified from Scognamillo et al., 2002, taken from Hoogesteijn & Hoogesteijn, 2014), below.

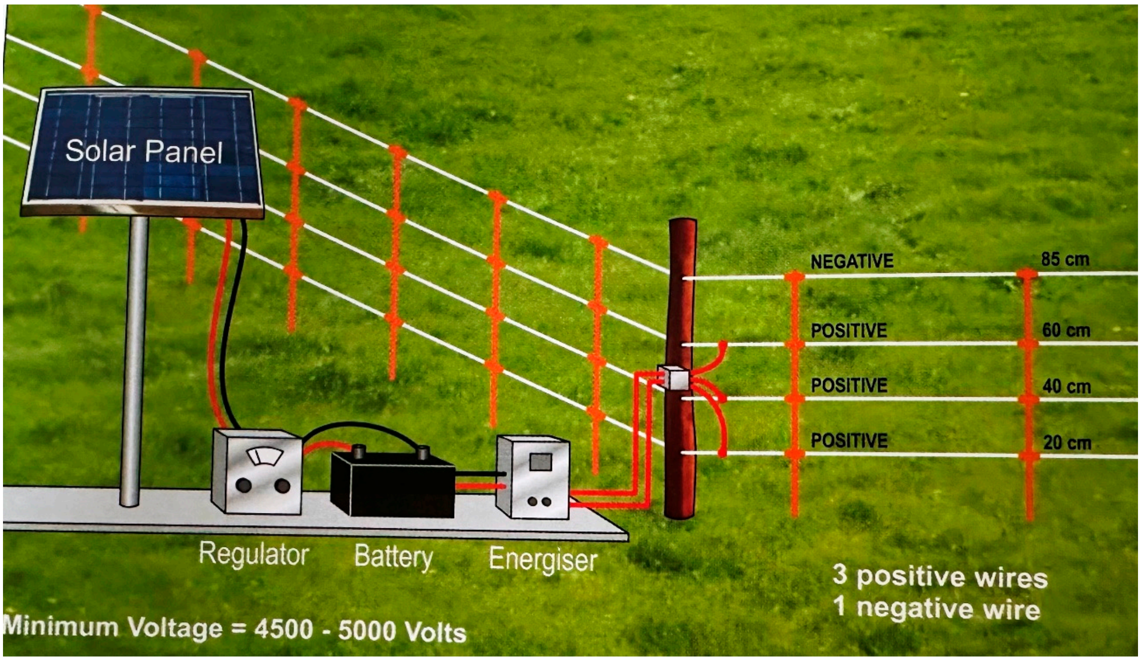

2B) APEF USED IN COLOMBIA, DIAGRAM 2:

In the Second Diagram, we observe the construction of the APEF we used in Colombia in the model livestock farms, in which Panthera Colombia and Webconserva worked (Valderrama et al., 2017, 2024; and this paper). The design is similar but can use 2, 3 or 4 strands of electrified wire and also incorporates a barbed wire at the lowest part, since in the Llanos of Casanare where most of the livestock farms that participated in these Feline / Livestock Conflict Control Programs were located; there is a certain abundance of land turtles (of the *Chelonoidis* genus), which cannot walk backwards, and with this barbed wire they can deviate laterally and look for other paths without receiving the pulses of the electric shock.

DIAGRAM 2: Electric fence design implemented and used by Panthera Colombia and WebConserva, to contain livestock, repel predators and prevent the discharge of electric pulses to land turtles (tortoises).

In this Diagram with 2 strands of electrified wire, it can also be built with 3 or 4 electrified wires. Diagram by Mariana Hoogesteijn. Source: Modified from: Valderrama et al., 2017; 2024, diagram below.

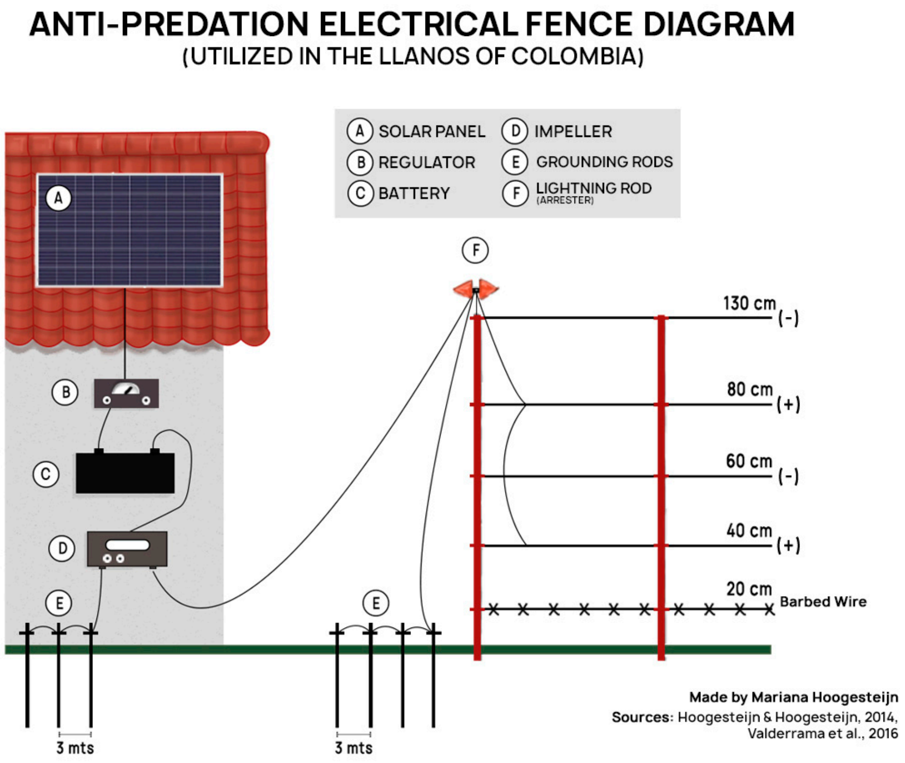

2C) APEF USED IN BRAZIL, DIAGRAM 3: In the third Diagram we observe the APEF structure that we developed at Panthera Brazil together with the technicians from Datamars – Speedrite (APEF devices and equipment) and from Belgo-Bekaert (for the wires used), in both cases recommended as quality equipment with good long-term performance (as a collaboration program without any commercial interest). For the lowest two strands of wire, we recommend high zinc-content wires to prevent oxidation / rusting in humid / flooded savanna conditions.

This type of APEF uses 2 or 3 strands of electrified wire, with only 2 positive strands (at 25 and 75 cm from the ground), as seen in Diagram 3 - when cattle herds are tame, and an additional positive wire that can be placed 1.25 m from the ground, if desired.

DIAGRAM 3:
Electric fence design implemented and used by Panthera Brasil in collaboration with Datamars-Speedrite and Belgo-Bekaert, to contain livestock and repel predators. In this case, with 2 strands of electrified wire, it also can be built with 3 electrified wires. Diagram by Mariana Hoogesteijn, Source: Modified from: Hoogesteijn and Viana, 2021; Hoogesteijn et al., 2024m below.

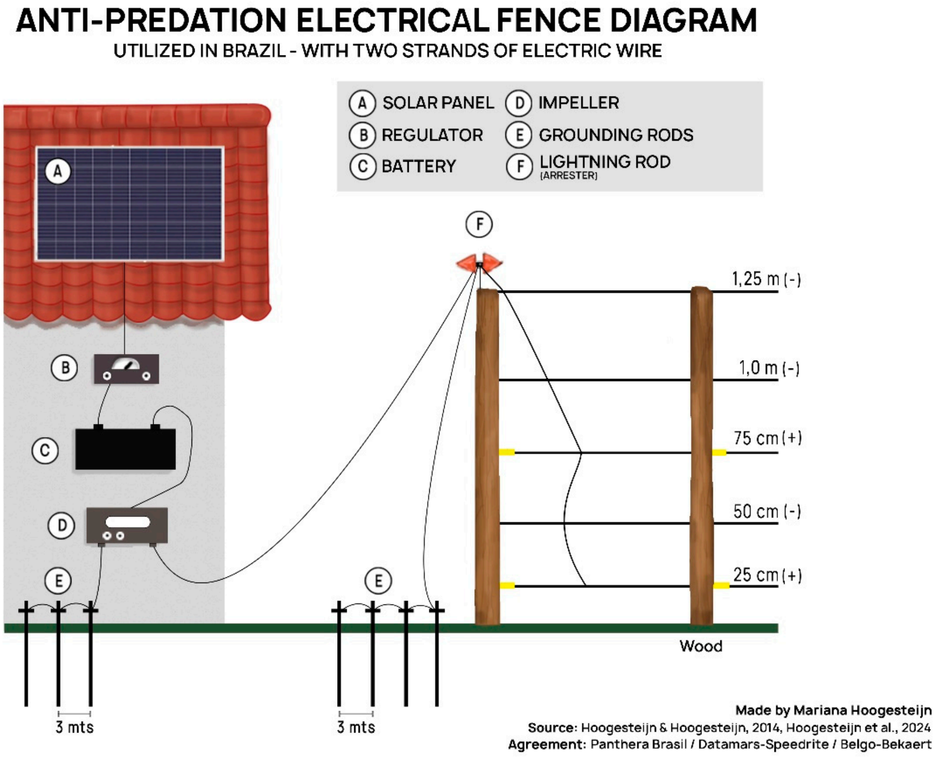

This is a structure of lower cost that works very well in Brazilian field conditions. It is important to note the quality of the insulators, which must be UV treated, otherwise after only 2-3 years of use, they begin to crack and leak energy, requiring the costly replacement of the insulators throughout the whole fence to solve this problem.

3) IMPORTANT POINTS TO TAKE INTO ACCOUNT IN THE CONSTRUCTION AND MAINTENANCE OF APEF:

A. Use top quality materials both in the construction and in the quality of the wires and equipment of the APEF itself. The wire (of good quality), must be well stretched between the poles; and the use of the lightning arrester and bronze poles (or grounding rods) as negative grounding are essential. The lightning rod protects the APEF and all the equipment from electrical discharges and the grounding with the grounding rods, guarantees that the electrical shock or impulse that both the feline that wants to enter and the animal that wants to leave, will be strong enough to contain it. This is especially true during the dry season when the negative conduction of the earth is minimal due to dry soil conditions. Therefore, in addition to the 3-6 grounding rods placed next to the lightning arrester, at least one grounding rod, must be buried and connected to the APEF negative wires every 500m - 1km, to achieve a satisfactory repellent effect.

B. The APEF check once in operation must be carried out at least weekly (better daily or 2-3 times a week) using the voltmeter, checking the charge of the positive wires, which should be above 5,000 Volts (better if it is between 7-8,000 Volts) to ensure a good repellent effect. It is essential to check the entire length of the APEF(s), especially the lower thread so that there are no tree branches, bushes or grasses that may be making contact and draining the energy flow of the system, thereby losing its repellent effect. During the dry season, maintenance is easier, as there is little vegetative growth of grass under the fence. But in the rainy season, regular cleaning is very important, at least every 2-4 weeks, keeping the lower part of the fence free of weeds first by manual

cleaning (with a machete or grass cutter) and/or chemical cleaning (afterwards) by applying a line of herbicide on the ground under the fence.

C. The fence cannot have openings through which a feline can sneak in. There cannot be tree branches that pass over the APEF that can allow the feline to jump into the enclosure / paddock. All openings and / or access gates & doors, must be electrified, or protected with steel mesh, so that the feline cannot enter through them.

The first three wire strands on the underside of the fence are essential to keep out predatory felines (1st at 25 cm positive, 2nd at 50 cm negative, and 3rd at 75 cm positive), as they try to sneak in, between the ground and the 1st wire, or between the 1st, 2nd and 3rd wires, in a stealthy manner. They generally do not jump over the fence, since that would alert their possible prey, although there are very rare cases in which they can learn to do so, or try to get out, once they entered inside through a defective part of the APEF, and do not find this same entrance to get out again. An alternative arrangement in sites subject to floodings or with land turtles that could be higher than 25cm in height, the first strand should be negative (1st at 25 cm negative, 2nd at 50 cm positive, and 3rd at 75 cm negative),

D.

APEF have proven to be a highly effective strategy for controlling (up to 100% on small properties) feline predation problems, but on the one hand, they are like a living organism, requiring loving care, attention and maintenance to function well, and the greater the area/perimeter/length/size of the APEF, the greater and better its maintenance must be. On the other hand, they allow to control predation losses to nil or tolerable levels, so the farmer/rancher has his ease of mind enhanced income, and the unpayable sense of sleeping at night without the stress of knowing that his (her) livestock can be exposed to feline predation anytime.

4) ADDITIONAL NOTES ON THE USE OF LED LIGHTS, FOX-LIGHTS AND APEF:

These lights have been shown to be very effective in several tests already carried out (e.g. Villalba et al, 2016, De La Torre et al., 2021) and recent ones (this paper), whether placed in night-time enclosure pens, small or medium-sized paddocks, or placed on collars made of luminous material together with bells (Corrales et al., 2016 a and b, https://pantherabr.com.br/).

However, effectiveness can vary in the sense that in areas in which there are jaguars habituated to tourism and human presence and that are less skittish, its effect can be shorter-term and the repellent effect, instead of lasting 2-3 years, may last only a few months.

Felines are very intelligent and once they have received a fence electric shock they will withdraw from the area for a few weeks and then display very cautious behavior, slowly approaching the fence wires again and detecting whether they are electrified or not. Likewise, over time they may detect that the lights do not have a direct physical repellent effect on their organisms and quite possibly will eventually end up ignoring them. Jaguars and pumas are very intelligent and clever, are always testing our defenses and trying to outsmart us, so the best thing (in addition to giving APS good maintenance), is to vary/rotate the APS over time, so that the cats do not get used to them.

5) REFERENCES:

Corrales-Gutiérrez, D, R. Salom-Pérez y R. Hoogesteijn. 2016-a. Implementación de estrategias anti-depredatorias en fincas ganaderas ubicadas dentro de dos importantes corredores biológicos de Costa Rica. En: Carlos Castaño-Uribe, Carlos A. Lasso, Rafael Hoogesteijn, Angélica Díaz Pulido y Esteban Payán-Garrido (Editores) II. Conflicto entre

Felinos y Humanos en América Latina. Serie Editorial Fauna Silvestre Neotropical. Instituto de Investigación de Recursos Biológicos Alexander von Humboldt, Fundación Herencia Ambiental Caribe, Panthera. Bogotá, D.C. Capítulo 9, Págs.: 152 – 167.

Corrales-Gutiérrez, D., R. Salom-Pérez y R. Hoogesteijn. 2016-b. Convenio entre el gobierno de Costa Rica y Panthera: Unidad de Atención de Conflictos con Felinos (UACFel). En: Carlos Castaño-Uribe, Carlos A. Lasso, Rafael Hoogesteijn, Angélica Díaz Pulido y Esteban Payán-Garrido (Editores) II. Conflicto entre Felinos y Humanos en América Latina. Serie Editorial Fauna Silvestre Neotropical. Instituto de Investigación de Recursos Biológicos Alexander von Humboldt, Fundación Herencia Ambiental Caribe, Panthera. Bogotá, D.C. Capítulo 10, Pags.: 169 – 180.

De La Torre J. A., G. Camacho, P. Arroyo–Gerala, I. Cassaigne, M. Rivero & A. Campos-Arceiz. 2021. A cost-effective approach to mitigate conflict between ranchers and large predators: A case study with jaguars in the Mayan Forest. Biological Conservation 256 (2021) 109066. https://doi.org/10.1016/j.biocon.2021.109066.

Hoogesteijn, R. and A. Hoogesteijn. 2014. Anti-Predation Strategies for Cattle Ranches in Latin America: A Guide. PANTHERA. Eckograf Soluções Impressas Ltda., Campo Grande, MS, Brazil. 64 pp. ISBN No. .978-85-912016-2-4.

Hoogesteijn, R; E. Payán Garrido, C. A. Valderrama Vásquez, A. Hoogesteijn, F. R. Tortato, R. Salom Pérez, D. Corrales Gutiérrez, e H. B. Quigley. 2024 (In Press). Protocolo para a Resolução de Conflito Felinos / Pecuária na América Latina. Secção I: Diretrizes de Diagnóstico e Operação (versão em Português). Panthera, PEX Soluções Gráficas, Campo Grande, MS, Brasil.131 pp.

Scognamillo, D., I. Maxit, M. Sunquist and L. Farrell. 2002. Ecología del jaguar y el problema de la depredación en un hato de los Llanos Venezolanos. En: R.A. Medellín, C. Equihua, C. Chetkiewicz, P.G. Crawshaw Jr., A. Rabinowitz, K.H. Redford, J.G. Robinson, E.W. Sanderson y A.B. Taber (Eds.). El Jaguar en el Nuevo Milenio. Fondo de Cultura Económica, Universidad Autónoma de México y Wildlife Conservation Society. México. pp. 139 – 150.

Valderrama Vásquez, C. A., R. Hoogesteijn y E. Payán Garrido. 2016. GRECO: Manual de campo para el manejo del conflicto entre humanos y felinos. Panthera y USFWS. Fernando Peña Editores. Cali, Colombia. 81 pp.

Valderrama-Vasquez, C.; R. Hoogesteijn, E. Payán, H. Quigley & A. Hoogesteijn. 2024. Predator-Friendly Ranching, use of Electric Fences, and Creole Cattle in the Colombian Savannas. European Journal of Wildlife Research 70:1. https://doi.org/10.1007/s10344-023-01754-3

Villalba, L.; L. Maffei, M. Fleytas & J. Polisar. 2016. Primeras experiencias de mitigación de conflictos entre ganaderos y grandes felinos en estancias de Paraguay. En: Carlos Castaño-Uribe, Carlos A. Lasso, Rafael Hoogesteijn, Angélica Díaz Pulido y Esteban Payán-Garrido (Editores) II. Conflicto entre Felinos y Humanos en América Latina. Serie Editorial Fauna Silvestre Neotropical. Instituto de Investigación de Recursos Biológicos Alexander von Humboldt, Fundación Herencia Ambiental Caribe, Panthera. Bogotá, D.C. Capítulo 14: Págs.: 227 - 236.
