# Supplementary material for: Sixty Degrees of Solutions: Field Techniques for Human–Jaguar Coexistence
Source: Animals (Basel). 2025 Apr 28;15(9):1247. doi: 10.3390/ani15091247 (PMC12071174; doi:10.3390/ani15091247)
Supplement: Supplementary file 1 [file animals-15-01247-s001.zip › S4. Broad Acknowledgements Team X Team 01122025.pdf]

Broader Acknowledgements team x team S4

Argentine Yungas: . The team from the Yungas in Argentina express gratitude to park rangers, volunteers and local people, the Comunidad Kolla El Lipeo y Baritú, Comunidad Kolla Finca Santiago, Administración de Parques Nacionales - Delegacióon Regional Noroeste (APN-DROA), Parque Nacional Baritú and Consejo Nacional de Investigaciones Científicas y Técnicas (CONICET).

Brazilian Atlantic Forest/Projeto Onças do Iguaçu: We are grateful to the local communities surrounding Iguaçu National Park for their invaluable partnership throughout this project. We also thank ICMBio for authorizing the research, as well as all the institutions and individuals who supported the project

Brazilian Pantanal - Fazenda Jofre Velho: Rafael Hoogesteijn would like to express his gratitude for the support of Alan Rabinowitz, Howard Quigley, Luke Hunter and Tom Kaplan of Panthera NY, and Dr. Luis A. Figueredo, owner of Fazenda Porto Jofre, on his initial proposal of creating Fazenda Jofre Velho and Panthera Brasil. We also thank the valuable assistance in the field help and technical contributions of Elizeu Evangelista da Silva, Ercio Evangelista da Silva†, Fernando Tortato, Mirtha Carpio, Raissa S. Alves, Eledilson de Sousa, Clarindo Canavarros, Esteban Payán, Elías Pelachim (Datamars), Guilherme Viana (Belgo) and other members of the Panthera Brasil – Fazenda Jofre Velho Team.

Brazilian Pantanal (Instituto Homem Pantaneiro): Thanks to the team of collaborators and volunteers from the Instituto Homem Pantaneiro, residents of riverside communities, Panthera Brasil, and the Serra do Amolar Protection and Conservation Network.

Brazilian Varzea (flooded forest); Local communities of the Mamirauá Reserve, field and research assistants, Members of the Ecology and Conservation of Felines in the Amazon Research Group, logistic and administrative support provided by the employees of the Mamirauá Institute for Sustainable Development, Integrative Conservation PhD Program & Warnell School of Forestry and Natural Resources-University of Georgia.

Colombian Llanos: We would like to express our gratitude to the Red de Reservas de Altagracia, local guides, Junta de Acción Comunal (JAC) of La Chapa, Matapalito, Porvenir de Guachiría, and Pirichigüa, cattle ranchers of Casanare, and CIPAV, as all as the ranch managers and staff and the teams of Panthera and WebConserva.

Costa Rican Tortuguero and Maquenque: We would like to thank the ranchers in the buffer zone of Tortuguero National Park, especially Wagner Duran and Danilo Campos, and from the Maquenque Mixed National Wildlife Refuge Minor Rojas and Kenneth Vargas, as well as the veterinarian Heiner Castro, the electric fence specialist Jesús Barrantes, and the researcher's right-hand man, Mr. Erick Víquez. Finally, we would like to thank USG, GOCR, CI, TNC, the Costa Rica Forever Association, the National System of Conservation Areas (SINAC), and the Feline Conflict Response Unit (UACFel / SINAC-Panthera-Corfoga-HSI).

French Guiana – HISA : The HISA team expresses its sincere gratitude to the farmers collaborating in this project, and the Office Français pour la Biodiversité (OFB), and to the agricultural cooperatives, particularly SCEBOG, and the KWATA team for their invaluable advice and support.

Mexico north (Sonora): Ron Thompson, Jesus Moreno Martínez, Berenice Portillo, Antonio de la Torre, Francisco Abarca, Anne Justice-Allen, Azucena Legorreta, Nick Smith, Dana Melani, Bert Geary, Kyle Thompson and the Southwest Center for Wildlife Conservation.

Mexico south (Selva Lacandona - Jaguares de la Selva Maya Program): The team is very grateful to the following communities of the Selva Lacandona region for their support and commitment to jaguar conservation: Boca de Chajul, Playón de la Gloria, Galacia, Flor de Marqués, El Pirú, San Isidro, Adolfo López Mateos, Quiringuicharo, Pico de Oro, La Corona, La Victoria & Reforma Agraria

Nicaraguan Mosquitia: Gobiernos Indigenas territorial/indigenous territorial governments of the Regimen Especial Alto Wangky y Bocay (Upper Coco and Bocay), Mayangna Sauni Bu, Kipla Sait Tasbaika, Miskito Indian Taskbaika Kum

Paraguay: To the owners of Estancias Ganaderas del Chaco Paraguayo - Estancia Madrejon, Campo Grande, La Ñeca and Campo 13 for working with, WCS to be part of the solution.

Venezuela: The research team in Venezuela would like to express their gratitude to the management of Desarrollos Forestales San Carlos, S.A. (DEFORSA) and to the non-government organization SEBRABA Project.
